# Occipital-temporal cortical tuning to semantic and affective features of natural images predicts associated behavioral responses

**DOI:** 10.1038/s41467-024-49073-8

**Published:** 2024-07-09

**Authors:** Samy A. Abdel-Ghaffar, Alexander G. Huth, Mark D. Lescroart, Dustin Stansbury, Jack L. Gallant, Sonia J. Bishop

**Affiliations:** 1 Department of Psychology, UC Berkeley, Berkeley, CA 94720 USA; 2 Google LLC, San Francisco, CA USA; 3 Centre for Theoretical and Computational Neuroscience, UT Austin, Austin, TX 78712 USA; 4 Department of Psychology University of Nevada Reno, Reno, NV 89557 USA; 5 Program in Vision Sciences, UC Berkeley, Berkeley, CA 94720 USA; 6 Helen Wills Neuroscience Institute, UC Berkeley, Berkeley, CA 94720 USA; 7 School of Psychology, Trinity College Dublin, Dublin, Ireland; 8 Trinity College Institute of Neuroscience, Trinity College Dublin, Dublin, D02 PX31 Ireland

**Keywords:** Emotion, Cognitive neuroscience

## Abstract

In everyday life, people need to respond appropriately to many types of emotional stimuli. Here, we investigate whether human occipital-temporal cortex (OTC) shows co-representation of the semantic category and affective content of visual stimuli. We also explore whether OTC transformation of semantic and affective features extracts information of value for guiding behavior. Participants viewed 1620 emotional natural images while functional magnetic resonance imaging data were acquired. Using voxel-wise modeling we show widespread tuning to semantic and affective image features across OTC. The top three principal components underlying OTC voxel-wise responses to image features encoded stimulus animacy, stimulus arousal and interactions of animacy with stimulus valence and arousal. At low to moderate dimensionality, OTC tuning patterns predicted behavioral responses linked to each image better than regressors directly based on image features. This is consistent with OTC representing stimulus semantic category and affective content in a manner suited to guiding behavior.

## Abstract (web summary)

The mechanisms of recognition and response to emotional stimuli are not fully understood. Here, the authors reveal tuning to semantic and emotional image features within occipital temporal cortex that efficiently encodes information suited to guiding behavior.

## Introduction

The ability to recognize and respond appropriately to emotionally salient stimuli is essential to a species’ evolutionary success. Adaptive responses to dangerous situations carry a survival advantage, while identification of potential mates and protection of offspring facilitates reproductive success. Optimal behavior is likely to extend beyond the simple choice to approach or avoid and to entail selection from a complex range of alternate behaviors^1^. For example, encountering a large bear versus a weak, diseased, animal should promote different types of avoidance responses, while conspecific infants and potential mates should prompt different types of approach response. We know relatively little about the neural mechanisms that facilitates these types of behavioral choices. Activation to emotional stimuli has been reported across a variety of brain regions including the amygdala, medial and lateral regions of frontal cortex as well as occipital and superior temporal cortex^2–11^. Here, we focus on the potential functional role of occipital temporal cortical regions in the representation of, and response to emotional natural stimuli. Within occipital temporal cortex, different sub-regions have long been known to preferentially respond to various subcategories of animate and inanimate stimuli, including faces, bodies, places and objects^12–18^. In recent years, it has been proposed that differential representation of distinct categories of objects and scenes in ventral occipital areas might be linked to their function and behavioral affordances as well as to their visual form^19–21^. This has been supported by findings from multi-modal object recognition studies and categorization studies in congenitally blind participants^19,20,22^. Importantly, however, much of this work has largely focused on inanimate stimulus categories such as tools or navigable locations^21,23,24^.

If behavioral affordances inform representation of objects and scenes in OTC, integrated representation of stimulus semantic and affective features might arguably be a logical sequala. For example, if we encounter a snake or cockroach in our vegetable garden, it might be beneficial not only to register a negative affective response but to access semantic information linked to the category of species encountered, for example, as to whether it might be poisonous (reptiles) or disease-carrying (insects). This in turn might facilitate our response. We hence set out to investigate whether there is evidence for integrated representation of stimulus semantic category and affective features within occipital temporal cortex and to test whether the information represented might be suitable for guiding behavioral responses. Finally, we were also interested in whether co-representation of semantic category and affective features varies as a function of stimulus animacy.

To address these questions, we adopted a voxel-wise multi-feature encoding model framework developed within the visual neuroscience literature^25–28^. Used in conjunction with functional magnetic resonance imaging (fMRI), presentation of a large diverse emotional natural image stimulus set, and acquisition of participant-specific ratings of stimulus valence and arousal, this modeling framework enabled us to investigate tuning to image semantic category and affective information at a single voxel level across OTC. Our findings revealed representation of natural image semantic category and affective features across much of OTC. Principal components analysis of voxel-wise feature weights indicated that many OTC voxels are co-tuned to semantic and affective information. Considering the first three components alone, we found co-representation of image animacy and valence as well as image animacy and arousal. Scores on these three components explained 20% of the variance in novel subjects’ selection of behavioral responses to match image content (e.g. the response ‘retreat from’ might be selected for an image of a snarling dog). Using scores on the first twenty components explained 40% of variance in novel subjects’ selection of behavioral responses across images. This prediction of behavioral responses from OTC components based on tuning to both semantic category and affective image features significantly exceeded that achieved using OTC tuning to low-level structural image properties or semantic category or affective features alone. It also significantly exceeded the variance explained by components from PCA performed directly on stimulus features, across images viewed. This is consistent with OTC showing augmented tuning to those image semantic and affective properties that are of value to guiding behavior.

## Results

### Using a multi-feature encoding modeling approach to investigate representation of natural image semantic and affective features

**Fig. 1:**
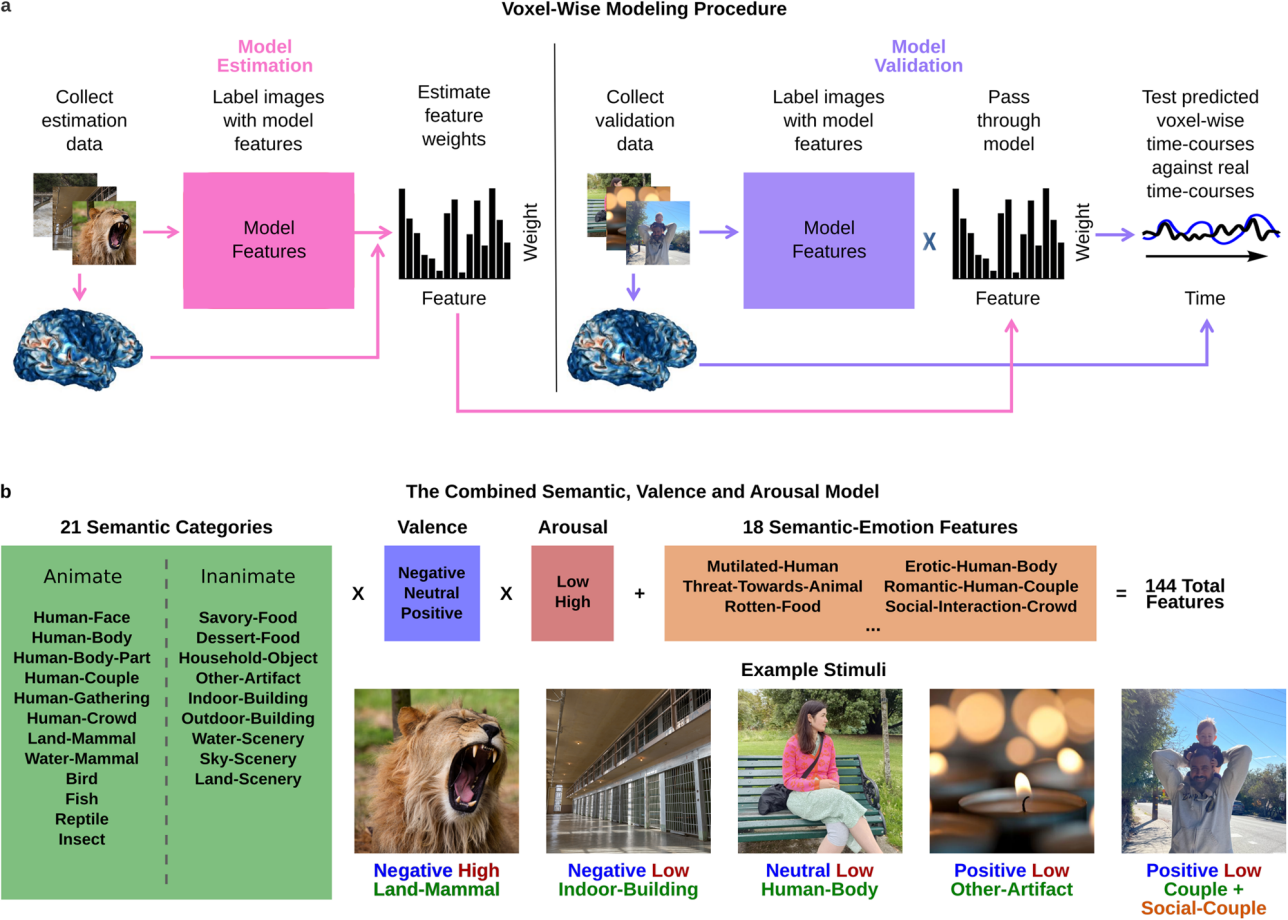
The modeling procedure and the CSVA model. **a** BOLD data collected while subjects viewed 1440 images were used for model estimation. Ridge regression was adopted to fit each model to the BOLD time-series for each voxel, using a finite impulse response function with four 2 s time-bins. Weights were estimated for each model feature for each time-bin. These weights characterize each voxel’s response profile or ‘tuning’ to model features. Model validation was conducted using independent fMRI data collected while subjects viewed 180 novel images. Voxel-wise feature weights were used to generate a predicted time-series for each voxel. This was correlated with the recorded BOLD time-series to obtain a metric of model fit that controls for over-fitting, see Methods for details. **b** The Combined Semantic, Valence and Arousal (CSVA) model comprises 126 mutually exclusive features denoting image semantic category (21 categories), valence (3 levels) and arousal (2 levels) and 18 additional semantic-emotion (SE) compound features that carry both semantic and affective information (e.g. mutilated human; rotten food), see Methods for further details. Image semantic category and SE features were labeled by four independent raters; image valence and arousal were assessed by each subject, see Methods for further details. Here, five example images are labeled with CSVA features. Due to copyright reasons, the images from our database have been replaced with similar images where the photographer, and subject when relevant, have provided consent for the image to be used and publically shared. See table S4 for source & licensing details.

Our experimental stimuli comprised 1620 images varying widely in semantic category and affective content. We labeled each image for features of interest (see Methods for details). Using ridge regression, we fit multi-feature encoding models to the functional magnetic resonance imaging (fMRI) data acquired while subjects viewed the images (see Methods and Fig. 1a). We tested alternate models by changing the features included as regressors. To have sufficient statistical power to fit the multi-feature encoding models used required the acquisition of a large amount of fMRI data per subject. The six subjects in our study each completed fifty fMRI scans across six 2 h sessions. Thirty training scans, each 7.5 min long, were used for model estimation, and twenty test scans, each 6 min long, were used for model validation. Images were presented at the center of the screen and viewed for 1 s each, with a 3 s inter-stimulus interval. Subjects either categorized the valence (negative, positive, neutral) or the broad semantic category (human, animal, food, object, building /scene) of each image. We used these two different tasks to ensure that cortical tuning to stimulus affective features was not only observed when task-relevant^29,30^. Subjects who performed the semantic categorization task in the scanner subsequently categorized the images by valence in a post-scan behavioral session. All subjects also rated the images for emotional arousal in this post-scan session (see Methods for details and Fig. S1 for the percentage of images falling within each valence by arousal bin for each participant).

### Mapping tuning to semantic and affective image features across cortex

**Fig. 2:**
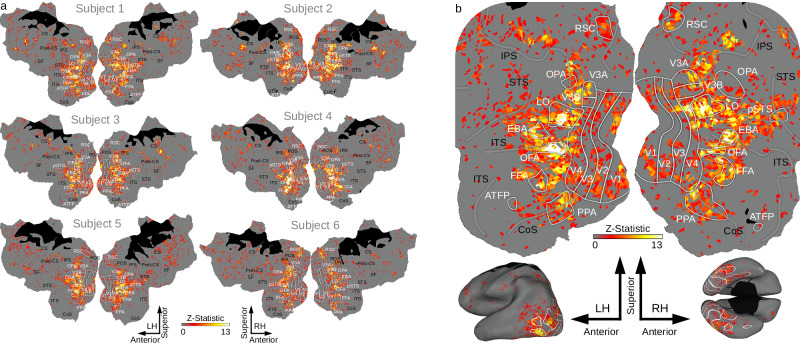
CSVA model voxel-wise prediction accuracy scores mapped onto cortex. **a** Voxels where activity was significantly predicted by the Combined Semantic, Valence and Arousal (CSVA) model are shown on cortical maps for all 6 subjects. For each voxel, prediction accuracies were calculated using the z-transformed correlation between the CSVA model predicted time-course and the recorded BOLD time-course for the validation dataset. Significance was assessed by permutation testing, see Methods for details. The CSVA model significantly predicts validation BOLD time-courses across much of OTC. This was consistently observed across subjects. **b** Prediction accuracy scores for subject 1; the cortical map is cropped (top) to zoom in on OTC. In addition, prediction accuracy scores are projected onto inflated lateral (bottom left) and ventral (bottom right) cortical surfaces. Note: Regions of interest (ROIs) are labeled in white, sulci in black. RSC Retrosplenial Complex, OPA Occipital Place Area, LO Lateral Occipital cortex, pSTS Posterior Superior Temporal Sulcus, EBA Extrastriate Body Area, OFA Occipital Face Area, FFA Fusiform Face Area, PPA Parahippocampal Place Area, ATFP Anterior Temporal Face Patch. IPS Intraparietal Sulcus, STS Superior Temporal Sulcus, ITS Inferior Temporal Sulcus, CoS Collateral Sulcus.

Our primary model, the Combined Semantic, Valence and Arousal (CSVA) model (Fig. 1b), describes each image using a combination of mutually exclusive semantic categories, subjects’ subjective valence and arousal judgments, and a number of additional semantic-emotion (SE) compound features conveying both semantic and affective information (e.g. ‘rotten food’, ‘mutilated human’), see Methods for details. FMRI data from the model estimation runs were concatenated and ridge regression used to fit the CSVA model to each subject’s BOLD data using a finite impulse response function with four 2 s time-bins. Voxel-wise weights were estimated for each model feature for each time-bin. These weights were applied to the values of feature regressors for the images viewed during the validation scans, generating predicted BOLD time-courses for each voxel. We correlated these predicted time-courses with the observed validation BOLD time-courses to obtain estimates of model prediction accuracy for each voxel (see Methods for further details). Figure 2a shows the resulting prediction accuracies projected onto the cortex of each individual subject. These cortical maps reveal that the CSVA model significantly predicts validation BOLD time-courses across a wide stretch of OTC. Figure 2b shows a close up of OTC for one example subject. Figure S2 shows the areas of cortex where the CSVA model outperforms a Gabor model which captures low level differences in image structure. It can be seen that the latter performs better in V1-V4 but the CSVA model outperforms the Gabor model outside of these retinotopic visual areas. As an additional control analysis, we regressed out variance in the BOLD data that could be explained by respiration or pulse-oximetry measures (see Supplementary Information). There was minimal change in prediction accuracies (Fig. S3).

In principle, the CSVA model could significantly fit subjects’ BOLD data simply as a result of BOLD responses to image semantic or affective features alone. One of the advantages of the voxel-wise modeling framework employed is that this can easily be explored. Specifically, we compared the fit of the CSVA model against that of a model containing only the semantic category features from the CSVA model (the Semantic Only model) as well as against that of a model containing only the valence and arousal features (the Valence by Arousal model). Each of these simpler models was fit to the estimation data, the validation data was then used to calculate voxel-wise prediction accuracies in the same manner as for the CSVA model. A bootstrap procedure was used to compare CSVA model fit against that of each of the other two models (see Methods for details). Model comparison was restricted to voxels where at least one of the three models showed a significant fit (see Table S1 for voxel counts per subject). Results revealed that the CSVA model outperformed the Valence by Arousal model at a group level and for all six subjects considered individually (*ps* < 0.05), Fig. S4. The CSVA model also outperformed the Semantic Only model at the group level and for four out of six subjects considered individually (*ps* < 0.05). CSVA model superiority to the Semantic Only model was apparent in stretches of OTC adjacent to, and overlapping with, regions with known semantic selectivity including the Occipital Face Area (OFA), Fusiform Face Area (FFA), posterior superior temporal sulcus (pSTS) and extra-striate body area (EBA), Fig. S5.

A convergent approach to the model comparison procedure described above is provided by variance partitioning. This technique estimates the variance explained by different elements of the CSVA model for each voxel (see Supplementary Methods: Variance Partitioning). Figure S6 shows CSVA model prediction accuracies for each subject after variance explained by semantic category features or affective (valence by arousal) features is partitioned out. It can be seen that many of the voxels responsive to the full CSVA model still show significant prediction accuracies when only variance explained by semantic category by affective feature interactions and the influence of modifiers (rotten, mutilated etc.) used to create compound semantic-emotion (SE) features is retained.

**Fig. 3:**
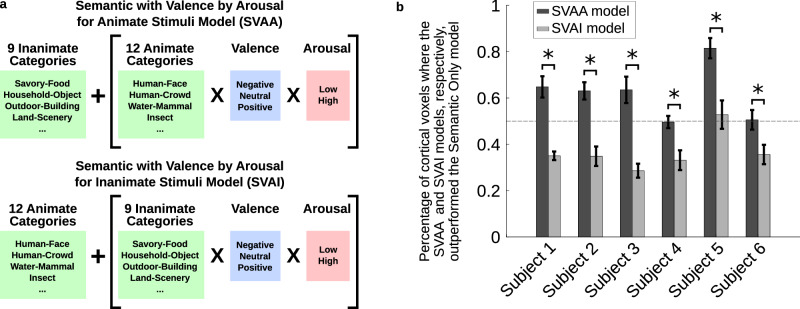
Cortical tuning to stimulus affective content is greatest for animate stimuli. **a** We examined the extent to which including the valence and arousal of either animate or inanimate stimuli improved model fit over and above modeling semantic category information alone. To investigate this, we constructed two additional models. The Semantic with Valence by Arousal for Animate Stimuli (SVAA) model includes features for each semantic category, but only stimuli belonging to animate semantic categories are also labeled for valence and arousal. The Semantic with Valence by Arousal for Inanimate Stimuli (SVAI) model includes features for each semantic category, but here only stimuli belonging to inanimate semantic categories are labeled for valence and arousal. **b** Model comparison was performed using the same cortical mask as for comparison of the CSVA model against the Semantic Only and Valence by Arousal models. This plot shows the percentage of voxels where the SVAA and SVAI models, respectively, outperformed the Semantic Only model, +/- SEM calculated across bootstrap samples. Data are presented separately for each individual subject. The number of voxels included in this analysis were as follows: subject 1 *n* = 6777, subject 2 *n* = 6450, subject 3 *n* = 5483, subject 4 *n* = 6596, subject 5 *n* = 6580, subject 6 *n* = 6618 (see Methods and Table S1). For all six subjects, labeling valence and arousal features for animate images improved model fit to a greater extent than labeling valence and arousal features for inanimate images (* significant at *p* = 0.05, two-tailed confidence interval test with SEM calculated via 1000-resample bootstrap test). Using separate estimation and validation datasets penalizes the inclusion of additional regressors that capture noise as opposed to genuine signal in the data. This is likely to explain the poorer performance of the SVAI model relative to the Semantic Only model.

We further examined whether coding stimulus affective features differentially improved model fit for animate versus inanimate stimuli. This enables us to address the question of whether co-tuning to image semantic and affective features in OTC is observed for animate stimuli only, or both for animate and inanimate stimuli. We investigated this by adapting the Semantic Only model such that either animate semantic category regressors or inanimate semantic category regressors were replaced by regressors coding for semantic category, valence and arousal, Fig. 3a. The same cortical mask was used as for comparison of the CSVA model against the Semantic Only and Valence by Arousal models, as described above. Across all participants, we observed a significantly greater increase in prediction accuracies relative to the Semantic Only model baseline when coding of affective features (i.e. participants’ subjective valence and arousal ratings) was added for animate as opposed to inanimate stimuli, Fig. 3b. Many of the voxels that showed this pattern were located within OTC, Fig. S7. Building upon these initial findings, we applied PCA to CSVA voxel-wise feature weights to further examine the structure of voxel-wise tuning to stimulus animacy, valence and arousal within OTC. We report these analyses next.

### PCA on CSVA model feature weights reveals consistent patterns of OTC tuning to stimulus animacy, valence and arousal across subjects

The voxel-wise modeling approach adopted enables us to go beyond simply assessing model fit and to interrogate voxels’ response profiles, i.e. their ‘tuning’, to different stimulus features. The CSVA model includes over 100 features. As a result, it is unwieldy to examine and interpret spatial patterns of tuning for each individual feature. Instead, we can seek to identify the main structure underlying similarities and differences in feature response profiles, across voxels. This can be achieved by applying principal components analysis (PCA) to model feature weights across a given set of voxels^28^.

**Fig. 4:**
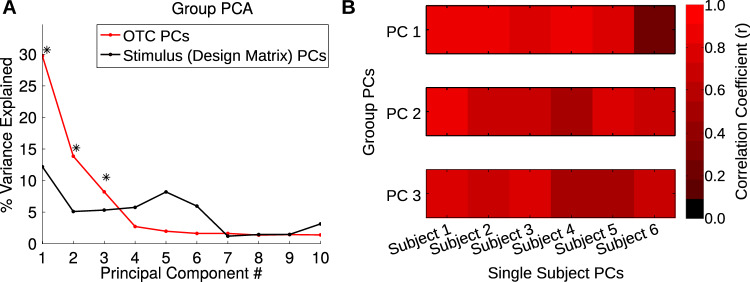
Results of PCA on CSVA model feature weights, across OTC voxels. **A** A group-level principal components analysis (PCA) was conducted on *CSVA* model feature weights across all OTC voxels where model fit was significant and better than that of the Semantic Only model. The scree plot shows the amount of variance explained by each of the top ten PCs (in red). PCs from a PCA analysis conducted on stimulus features (using the combined design matrix from all 6 subjects) are shown in black. Asterisks indicate group PCs that explain significantly more variance than the stimulus PCs (one-tailed jackknife test, *p = 0.03, this is the smallest possible *p* value given the jackknife test used), see Methods for details. **B** Results of a leave-one-out cross validation analysis of the similarity in feature loadings between individual subject PCs and group PCs. The correlation matrix presented gives the correlation of feature loadings for the top three PCs extracted from PCA conducted on each individual subject’s data and the top 3 PCs from the group-level PCA conducted on the data from all remaining subjects. All correlations shown are significant at *p* < 1E-8 (assessed by one-tailed permutation tests) except for subject 6 PC 1 where *p* = 0.0065, see Table S2). This indicates a shared representational structure across subjects.

We first conducted a group-level PCA on CSVA model feature weights across OTC voxels where the CSVA model fit significantly and predicted validation BOLD time-series better than the Semantic Only model, see Methods for details. (We note that expanding voxel selection to include all cortical voxels where the CSVA model fit significantly produced a highly similar PCA solution, *rs* > 0.95 for loadings of the top three group PCs, as did excluding voxels that fell within early visual cortex (EVC), rs>0.99 for loadings of top three group PCs, see Supplementary Information, Fig. S8, Fig. S9). Each of the top three PCs from the group-level PCA accounted for significantly more variance than could be explained by covariance between stimulus features alone (Fig. 4A), see Methods.

To examine consistency in PC loadings across subjects, we performed leave-one-out cross validation. Here, we conducted PCA on a given participant’s data and extracted the top three PCs. We then correlated the PC loadings, across features, with those of the top three PCs obtained from a group level PCA conducted with the data from the given participant left out. Significance was calculated via a permutation test (see Methods). This revealed that the top three PCs for each subject were significantly correlated with those of the remaining group indicating high consistency in the structure of OTC tuning to CSVA model features across subjects (Fig. 4B, Table S2). We note that this holds for all subjects, including subjects 2 and 4 who categorized images semantically while fMRI data were acquired (see Methods).

**Fig. 5:**
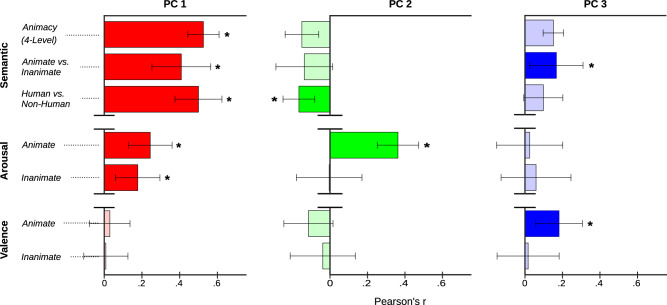
Top three dimensions underlying OTC tuning to semantic and affective image features carry information about stimulus animacy and its interactions with stimulus arousal and stimulus valence. A group-level PCA was conducted on CSVA model feature weights across OTC voxels (see Fig. 4). Feature loadings (*n* = 144) on the top three PCs were correlated, using Pearson’s r, with feature loadings on theoretical dimensions of interest. Bootstraping was used with 5000 resamples to perform one-tailed significance tests of correlation coefficients. Bars show Pearson correlation coefficients (r) +/- sd calculated from bootstrap samples for each of the top three PCs (left to right) against each theoretical dimension (y axis). Saturated color and * indicates correlations significant at *p* < 0.05, transparent colors indicate correlations that are not significant. PC1 carries information about stimulus animacy and the arousal value of both animate and inanimate stimuli. PC2 carries information about the arousal of animate stimuli; PC3 carries information about the valence of animate stimuli. PC2 and PC3 also show some tuning to animacy in general, though more weakly than PC1. Note. Theoretical dimensions: arousal is coded as high (+1) or low (0), valence is coded as positive (1), neutral (0), negative (−1), see Methods for details.

We explored OTC co-representation of stimulus animacy, valence and arousal by comparing the feature loadings of the top three group PCs to those of seven dimensions of theoretical interest (Fig. 5). The first of these ‘theoretical’ dimensions comprised a previously adopted 4-level index of animacy (human, other mammal, non-mammalian vertebrate/invertebrate, and inanimate^18^). The second comprised a binary index of whether image content was animate or inanimate. The third indexed whether humans were present in each image or not. The remaining four dimensions separately indexed the valence and arousal of animate and inanimate stimuli (see Methods for further details). We correlated the feature loadings of these theoretical dimensions with those of the three group PCs; this provides a measure of how well each theoretical dimension explains the information carried by each PC. Bootstrapping was used to determine correlation significance at *p* < 0.05 (see Methods for further details). This correlational analysis revealed that PC 1 represented both stimulus animacy and stimulus arousal (Fig. 5). PC 2 primarily represented the arousal value of animate, but not inanimate, stimuli. PC 3 represented the valence of animate, but not inanimate, stimuli. Both PC2 and PC3 also carried information about animacy in general, though more weakly than PC1.

**Fig. 6:**
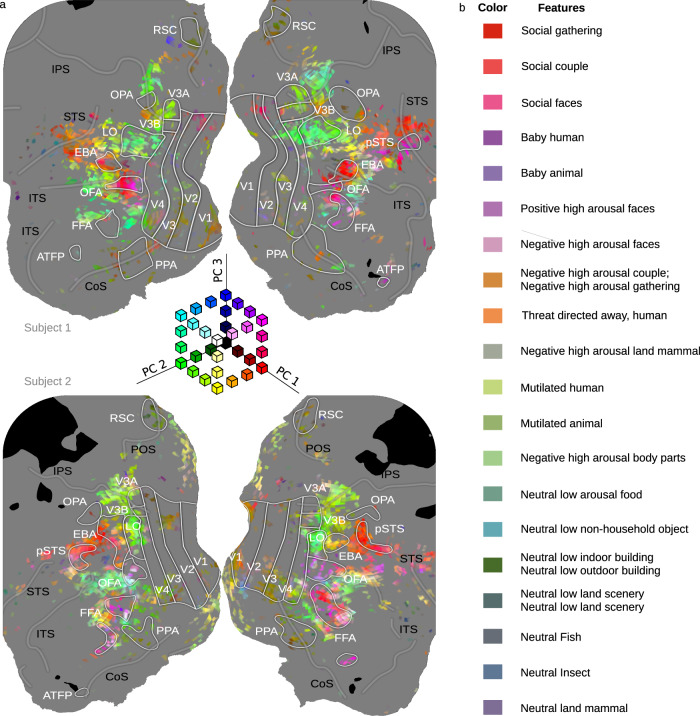
Structure of OTC tuning as captured by PCA on CSVA model feature weights. **a** Mapping PC scores from the group-level PCA of CSVA model feature weights, across OTC voxels, reveals considerable spatial structure in tuning to the top three PCs. Maps for two representative subjects (S1 and S2) are shown (see Fig. S10 for maps from all subjects). Voxel-wise PC scores were calculated as the product of CSVA model feature weights for a given voxel by feature loadings for each PC. A RGB color space is used; red=scores on PC1, green=scores on PC2, blue=scores on PC3. PC scores are thresholded at 6 standard deviations above and below 0, with values beyond the threshold given the maximal (or minimal) color channel value. Areas where MRI data were not acquired are shown in black. Both voxels where the CSVA model did not fit significantly and those where the CSVA model fit significantly but did not outperform the semantic only model were excluded from the PCA (these voxels are shown in gray). PCA maps using CSVA model feature weights from all voxels where the CSVA model fit significantly are given in Fig. S11. **b** Individual features are projected into the 3-dimensional space defined by the top three PCs, focusing on those parts of the space to which there is a strong response as shown in Fig. 6a. The first column ‘color’ shows the location in PC space using the same RGB color space as in Fig. 6a. The second column ‘features’ shows the features with loadings on the top three PCs that correspond to that location in PC space.

To visualize the spatial structure of tuning captured by these PCs, we projected voxel-wise PC scores onto maps of OTC for each subject (Fig. 6, Fig. S10, Fig S11). These maps show clear hemispheric symmetry in voxel-wise feature tuning across OTC; they also reveal commonalities in spatial transitions in tuning across subjects with distinct cortical patches responding selectively to particular parts of the space captured by the top 3PCs. To further explore this tuning, we projected individual features into the space defined by the top 3PCs. This projection is illustrated in Fig. 6b, here we focus on those parts of the PC space to which there is a strong response as shown in Fig. 6a.

**Fig. 7:**
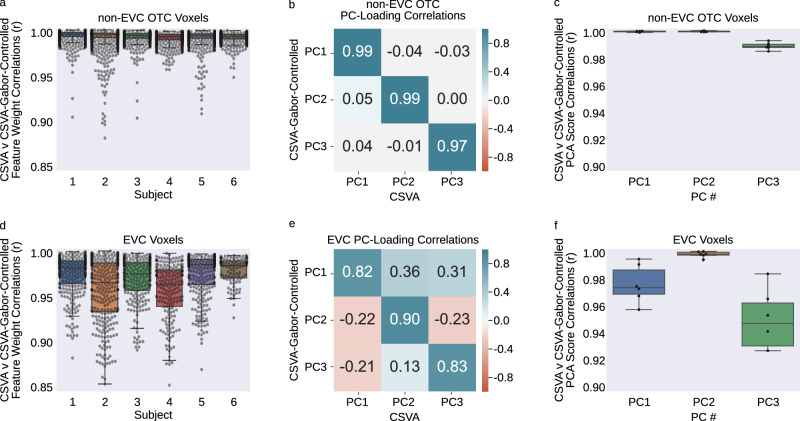
Correlations between Gabor and CSVA model features do not drive the variance captured by the CSVA model in OTC. Out-of-sample variance explained by the Gabor model was regressed out of the estimation BOLD data, and the residuals were used to re-fit the CSVA model (named CSVA Gabor-controlled) resulting in new feature weights. PCA was performed on these new weights. To clarify whether any changes in feature weights or PC loadings and scores were primarily observed in early visual cortex (EVC) or were also seen in non-EVC occipital temporal cortex (OTC), we divided our OTC ROI into these two regions (see Supplementary Methods). We conducted a separate PCA within each. **a**–**c** (top row) show findings for non-EVC OTC voxels; **d**–**f** (bottom row) show findings for EVC voxels. **a**, **d**. A bar and whisker plot shows voxel-wise correlations between feature weights from the CSVA and CSVA-Gabor-Controlled models for each subject across non-EVC OTC voxels and EVC voxels. Box plot elements: center line=median; box limits=upper and lower quartiles; whiskers=1.5 x inter-quartile range; individual points=feature weight correlation coefficients (across features) for each voxel. **b**, **e** A correlation matrix shows the similarity between feature loadings for the top 3 group PCs from the original CSVA model and the top 3 group PCs for the CSVA Gabor-controlled model for PCA conducted across non-EVC OTC voxels and EVC voxels. **c**, **f** A bar-and-whisker plot shows the correlations between PC scores from the original CSVA model and PC scores from the Gabor-Controlled model across non-EVC OTC voxels and EVC voxels. Box plot elements: as for panels **a** and **d** except individual points = PC score correlation coefficients (across voxels) for each subject.

We conducted a number of additional control principal components analyses. First, we sought to ensure that PC feature loadings did not simply reflect low-level image structure. To address this, we fitted a Gabor model to the estimation data and regressed out the variance explained before fitting the CSVA model to the residuals (see methods and supplementary information). PCA of the resulting CSVA model feature weights, across voxels, revealed minimal change in PC loadings in non-EVC OTC (*rs* > 0.97, see Fig. 7a–c). As expected, in EVC controlling for variance explained by the Gabor model changed CSVA feature weights and PC loadings to a somewhat greater extent (see Fig. 7d–f).

Given the role commonly ascribed to OFC in coding stimulus affective value^31–34^, we also conducted PCA of CSVA model feature weights across voxels in OFC and non-OFC frontal cortex (see supplementary information for frontal ROI definitions and PCA details). For each of these frontal regions, only the top two PCs showed good consistency across subjects (see Fig. S12) and there was much less consistent organization of the spatial structure of voxel-wise PC scores both within and across participants (Fig. S13, Fig S14). This is potentially consistent with theories according to which frontal cortical tuning shows high within and between subject flexibility in representation to take into account current task goals and changes in stimulus value across time and between contexts^29,30,32–35^.

### OTC tuning to emotional natural images predicts behavioral responses

**Fig. 8:**
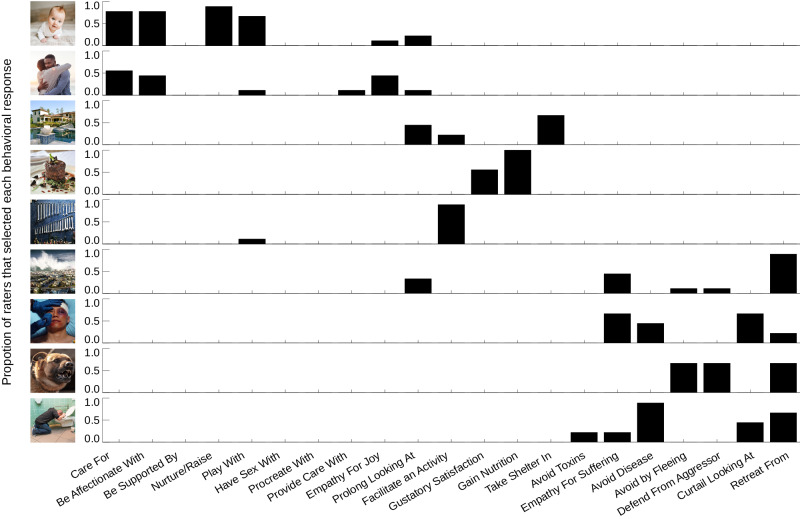
Behavioral responses matched to image content: example distributions. Subjects recruited through Amazon’s Mechanical Turk platform were shown the emotional natural images used in this study and asked to select one or more behavioral responses appropriate to the content of each image from a list provided. For each image, we calculated the proportion of MTurk subjects that selected each behavioral response. This is illustrated here for 9 example images^1^. The behaviors selected between are given on the x axis. Some terms have been abbreviated for illustration purposes (see Methods). In each row, the proportion of subjects selecting each behavior is plotted for a given image. ^1^Note, where images from our data-set did not have licences allowing for public sharing (including all images containing faces), they have been replaced by highly similar images with such licences in place. See table S4 for source & licensing details.

**Fig. 9:**
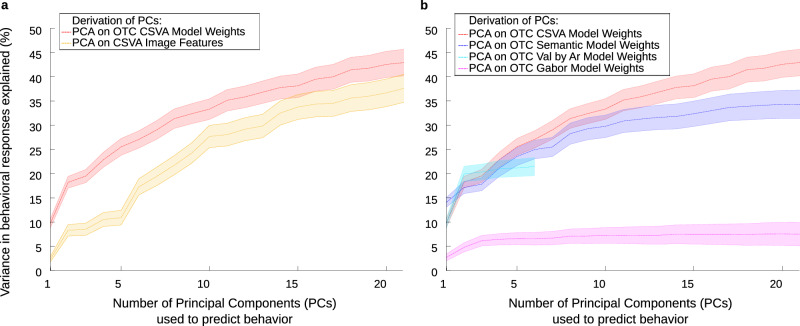
OTC tuning patterns predict behavioral responses to emotional natural stimuli. **a** We examined the extent to which OTC tuning to emotional natural images, as captured by CSVA group-level PC scores, predicted the behavioral responses selected for each image by MTurk raters, across images. The dotted red line represents the percentage of out-of-sample variance in behavioral responses explained as a proportion of potential explainable variance (y axis) plotted against the number of PCs included as predictors in the ordinary least squares regression analysis. The error band around the dotted line represents the 95% confidence interval (CI). We also calculated the scaled^1^ out-of-sample variance in behavioral responses explained using PCs derived directly from PCA on CSVA image features, across images (yellow dotted line; 95% CI is given by the associated error band). Across all levels of dimensionality considered (nu. of PCs=1 to 21), OTC tuning to CSVA features predicted behavioral responses significantly better than components from PCA conducted directly on the features themselves. This is consistent with OTC showing selective representation of image semantic and affective features pertinent to behavior. **b** Here, data are shown using the same format as in **a** Dotted lines represent scaled^1^ out-of-sample variance in behavioral responses explained by PCs obtained from PCA on OTC feature weights for the CSVA model (red) versus a Gabor model (pink), the Semantic Only model (dark blue) and the Valence by Arousal model (light blue). Error bands give the 95% CIs. Given the smaller feature space of the Valence by Arousal model, the maximum number of PCs that can be extracted for this model is six. The poor performance of Gabor model PCs in predicting behavioral responses suggests that OTC tuning to low-level image structural features is insufficient to guide behavior. Both the Semantic Only and Valence by Arousal models outperform the Gabor model in predicting behavior. However, their maximal prediction of behavior (at *n* = 21 and *n* = 6 PCs respectively) is significantly less than that achieved by the CSVA model using an equivalent number of components. ^1^Scaled by potential explainable variance (see methods).

Of central interest to us was the question of whether OTC tuning to the affective and semantic features of emotional natural visual images might be able to guide selection of behavioral responses in a manner that goes beyond a simple approach-avoidance dichotomy. To address this, we asked a separate set of subjects recruited via Amazon’s Mechanical Turk to select behavioral responses appropriate to the content of each image viewed by our fMRI subjects (see Methods for details). The frequency with which each image was associated with a given behavioral response was calculated (Fig. 8). We examined the extent to which OTC tuning to affective and semantic stimulus features, as captured by CSVA model group PC scores, predicted these behavioral responses, across images. We varied the number of PCs used from 1 to 21. We capped the number of components used to predict behavior at 21 to facilitate comparison with the Semantic Only model (given this latter model’s fewer features, this was the maximum number of PCs). Each image was given a score for each PC that represented the product of its features by feature loadings for that PC. These scores were used to predict out of sample behavioral responses across images (see Methods for details). The extent to which behavioral responses linked to each image were correctly predicted increased with the number of CSVA model PCs entered. In other words, as we included more dimensions of variance in OTC tuning to CSVA model features, we explained more variance in behavioral responses to the images viewed. The top 3 PCs alone accounted for 20% of explainable variance in behavioral responses across images, the top 10 PCs approximately 30%, and the top 20 roughly 40%. Notably, this performance was superior to that achieved using components from PCA conducted directly on CSVA features, across stimuli, Fig. 9a. This indicates that OTC organizes representation of stimulus semantic and affective features in a manner that increases mapping to potential behavioral responses at low to medium levels of dimensionality. This is consistent with the hypothesis that the structure of OTC tuning to semantic and affective features might be suitable to support selection between alternate approach and avoidance behaviors.

We next sought to determine whether OTC tuning to image semantic and affective features as captured by the CSVA model predicted behavioral responses significantly better than OTC tuning to low-level image structure. To accomplish this, we fit a Gabor model to OTC BOLD time-courses (see Methods for details), conducted PCA on Gabor model weights across significantly predicted voxels and used the top 1–21 of these PCs to predict behavioral responses to each image. Figure 9B shows that OTC tuning to emotional images as captured by Gabor model PCs explained significantly less variance in behavioral responses than that captured by CSVA model PCs (*ps* < 0.05). This indicates that OTC extraction of low-level structural image properties alone is unlikely to provide a basis for guiding behavior to emotional stimuli. Additional regression analyses revealed that the maximal performance of the Valence by Arousal model (at 6 PCs) and the Semantic Only model (at 21 PCs) was also significantly poorer than that of the CSVA model using an equivalent number of PCs, *ps* < 0.05, Fig. 9B. Here, as for the Gabor model, PCA was conducted on model weights across all OTC voxels where the model in question showed a significant fit. Taken together these results reveal that OTC representation of affective and semantic stimulus features predicts behaviors selected in response to the stimulus in question more efficiently than either the same information before transformation by OTC or OTC tuning to semantic category or affective information alone or to low-level structural features.

It has been argued that our responses to threat-related stimuli might be especially evolutionarily conserved. The information used to select between avoidance behaviors might hence differ in nature to that used to select between approach behaviors. We addressed this by conducting two further analyses that examined prediction of behaviors restricted to approach behaviors only or avoidance behaviors only. The results are given in Figure S15. Across the first ten principal components, OTC tuning to CSVA model features continued to explain more variance in behavioral responses than components from PCA conducted directly on CSVA features, this was seen all the way up to 20 components for approach behaviors. OTC tuning to CSVA models features also continued to perform better than OTC tuning to semantic categories only or to low-level structural features in predicting behavior. The one notable difference was that, for avoidance behaviors, OTC tuning to affective features alone (i.e. as captured by the valence by arousal model) substantially outperformed all other models in predicting behavior including OTC tuning to the full CSVA model.

As an additional control analysis, we excluded three of the response categories that arguably do not require explicit behavioral responses (be supported by, empathy for suffering, empathy for joy). Excluding these categories had minimal impact on the findings (Fig. S16). We also conducted two final analyses to quantify chance prediction performance (permuting behavioral responses across images) and to estimate an upper bound of prediction of behavioral ratings across images. For the latter, we conducted PCA on the behavioral ratings themselves to examine how much variance is explained by the first component, first two components etc (Fig. S17).

## Discussion

Evolutionarily, survival is linked to optimal matching of approach and avoidance behaviors to stimuli encountered in the world around us. Here, we considered the possibility that selection between such behaviors might be facilitated by combined representation of stimulus semantic information and affective value. We investigated whether human OTC shows such combined representation and if this representation might be of a nature able to guide behavioral responses to a wide range of natural emotional visual stimuli. Voxel-wise modeling of fMRI data collected while subjects viewed over 1600 emotional natural images revealed that many OTC voxels show combined representation of stimulus semantic category and affective value with this being most apparent for animate stimuli. A separate group of subjects selected behavioral responses that best matched image content. Regression analyses revealed that OTC tuning to semantic and affective stimulus features, as captured by PCA on CSVA voxel-wise model weights significantly predicted behavioral responses, across images. At low to medium levels of dimensionality, the amount of variance explained significantly exceeded that achieved when PCA was performed directly on semantic and affective stimulus features, across images viewed, i.e. before the information carried by these features was transformed by OTC. It also significantly exceeded that explained by OTC tuning to semantic category or affective features modeled alone as well as that explained by OTC tuning to low-level structural image features.

Within OTC, the presence of stretches of cortex with preferential tuning to different subtypes of animate and inanimate stimuli is well established^12–18^. It has been argued that representation of object and scene categories in OTC may carry information linked to stimulus behavioral affordances^19–21^. In addition, several studies have reported evidence that stimulus affective value is represented within OTC^8–11^. Here, we built on these literatures to ask whether there is corepresentation of stimulus semantic category and affective information within OTC and whether this representation is suited to enabling selection between alternate avoidance and approach behaviors. Our findings were consistent with co-representation of semantic category and affective information at a voxel-wise level within OTC, with coding of both stimulus valence and arousal for animate stimuli and some more limited coding of stimulus arousal for inanimate stimuli. Voxels sensitive to this information were found in stretches of cortex adjacent to areas with well-characterized selectivity to different categories of stimuli, notably neighboring FFA, EBA and pSTS. Within these well-characterized areas, semantic selectivity might have developed to facilitate navigation of our social environments. Our current results are consistent with selective tuning to semantic categories in adjacent regions being combined with tuning to stimulus affective information in a manner well-suited to guide behavioral selection.

More specifically, our findings suggest that OTC co-representation of image semantic category and affective properties has the potential to guide selection of diverse behavioral responses to a wide range of natural stimuli. Across the low to medium levels of dimensionality considered (1–21 PCs), OTC tuning to semantic and affective image features better predicted behavioral responses across stimuli than direct use of an equivalent number of dimensions based directly on the feature themselves (Fig. 9a). This potentially indicates that OTC is effectively compressing and extracting stimulus semantic and affective features of pertinence to behavior, i.e. representing these features in a more efficient fashion. Further, when a model of low-level visual structure, namely a Gabor model, was fitted to OTC voxel time-courses, it did a significantly poorer job than the CSVA model in predicting behavioral responses (Fig. 9b). This indicates that it is the encoding of higher-level features in this region of the brain, as opposed to lower-level structural features, that has the potential to be used to guide appropriate behaviors. Here, we note that controlling for low-level visual structure did not alter the structure of tuning to semantic and affective features within regions of OTC outside of early visual cortex (Fig. 7).

An important caveat is that we did not investigate mid-level structural features and cannot rule out that features such as stimulus color or shape might carry information about stimulus category or affective value that can be used to trigger behavioral responses. Indeed, it is possible that where we refer to encoding of semantic category, we might to some extent be capturing encoding of mid-level structural features that correlate with the stimulus categories used. This pertains to a wider debate on the representation of stimulus categories within OTC and could potentially be addressed in future work using mid-level structural models and stimulus sets designed to de-correlate mid-level structural and semantic information. For now, our findings indicate that OTC encodes representations well-suited to selecting between a range of alternate approach and avoidance behaviors in the context of emotional natural images where visual cues signal affective information which changes the optimal response to a stimulus falling within a given category.

It can also be argued that our main behavioral analysis demonstrates that OTC tuning to CSVA model features carries information able to guide selection between a mixed set of avoidance and approach behaviors but does not specifically address selection within avoidance behaviors and selection within approach behaviors. We addressed this through a further post-hoc analysis in which we subdivided behaviors into approach behaviors and avoidance behaviors. OTC tuning to semantic and affective image features, as captured by CSVA model PCs continued to predict behavioral responses better than either OTC tuning to semantic category alone or OTC tuning to image structure as captured by the Gabor model, Fig S15. However, OTC tuning to affective features alone (as captured by the Valence by Arousal model) performed best in prediction of avoidance behaviors. This finding might suggest that the relative importance of affective and semantic features differs between approach and avoidance behaviors, with affective stimulus features playing more of a key role in driving avoidance behaviors. We note, however, that this analysis was limited by there only being seven different avoidance behaviors. If a larger number had been used it is possible that we would have found the CSVA model to outperform the Valence by Arousal model.

As information reaches OTC rapidly^36^, representation of stimulus category and affective information within this part of cortex might be well suited to support behavioral responses to emotional stimuli. Here, we are not arguing that the amygdala is not involved in the initial learning of emotional value. Indeed, an interesting question would be whether insult to the amygdala early in development alters the nature of these OTC representations. One limitation of the voxel-wise modeling approach is that it is difficult to successfully fit amygdala activity. Prediction of the validation BOLD time series requires a consistent response to stimulus features across multiple presentations of the validation images. This reduces the ability to fit activity in regions sensitive to stimulus novelty that show non-stationary BOLD responses across presentations. We note that, ideally, a region informing choice between competing avoidance or approach responses to emotional stimuli should be able to give a consistent output despite multiple viewings of the stimulus in question. Hence, the method used is appropriate for identifying regions suited to consistently guiding selection of behavioral responses to emotionally salient stimuli.

Our findings reveal less clear spatial structure of tuning to semantic and affective stimulus information in frontal regions than in OTC (see Figs. S13, S14). We cannot rule out the possibility that reduced extent and spatial consistency of tuning to semantic and affective stimulus content in frontal regions might reflect lower sensitivity to detect such tuning as a result of differences in signal to noise across cortex. However, an interesting possibility is that the OTC might be particularly well suited to the long-term storage and rapid activation of the representations needed to select between alternate approach and avoidance behaviors. In contrast, tuning in frontal regions, might be flexibly reconfigured to suit current goals^29,30,35^. If this is the case, OTC and OFC might well play important complementary roles in guiding behavior. Evidence in support of this proposal has been provided by a number of studies examining the invariance of object representation to the precise task performed. Here, findings have consistent reported tuning in occipital-temporal regions to be more invariant to the precise task than tuning in frontal regions^29,30^. Intriguingly, in a study using a dynamically adaptive imaging procedure, Cusack and Mitchell also reported that OTC tuning to the animacy, valence and arousal of target items (a knife or dolphin) was maintained across task conditions (perception and imagery) to a greater extent than tuning to low-level structural features^37^.

An alternate fMRI analysis approach used in the field is a form of multivariate pattern analysis (MVPA) known as representational similarity analysis (RSA). Prior work using Representational Similarity Analysis (RSA) has failed to find interactions of stimulus valence and animacy in OTC^7^. The current study has greater within-subject statistical power than previous studies due to the collection of more BOLD data per participant. Both the difference in power and difficulties inherent in using RSA to study interactions (see Supplementary Information: RSA) might explain the discrepancy in results. While respecting the limitations of RSA, we also recognize the value of enabling readers to compare across studies. Hence, we conducted RSA on our current data-set. The findings are consistent with those from our primary analyses, revealing animacy by valence and arousal interactions in OTC (Figs. S18–S20).

Our current study did not set out to explore representation of internal emotional states provoked by the stimuli viewed or to shed light on whether neural representation of emotional states is more consistent with dimensional or categorical models of emotion. We asked participants to judge the valence and arousal of the stimuli viewed as the valence by arousal model is a mainstream model for representing stimulus affective value. Recent work has indicated that OTC tuning to movies and images can differentiate stimuli according to the emotion they provoke^8,10^. Notably, Kragel and colleagues developed a convolutional neural network model able to differentiate video clips evoking 11 distinct categories of emotions^8^. They reported that occipital cortical responses to the video stimuli were better able to differentiate these categories than other regions of cortex. Interestingly, in behavioral work with another stimulus set, they demonstrated that the output layer of their model was able to predict ratings of valence and arousal. The authors argued that these judgments might be emergent properties of the more detailed categorical emotion representations. In future work it would be of interest to determine the extent to which the combined semantic and affective features captured by our CSVA model map onto the final layer representations of the model used by Kragel and colleagues and to also evaluate the mapping between discrete emotional states and behavioral responses evoked by complex affectively charged natural images.

In summary, our findings are consistent with the co-representation of stimulus semantic category and affective information in OTC. This is observed in areas of cortex adjacent to, and partially overlapping with, areas with known selectivity to different categories of stimuli. Our analyses further indicate that tuning to semantic category and affective image features within OTC might carry information sufficient to drive rich behavioral responses to a diverse range of emotional natural visual stimuli. Indeed, the more efficient prediction of behavioral responses from OTC tuning to stimulus features than from the stimulus features themselves possibly indicates that OTC is effectively compressing and extracting information about semantic category and affective image content that is most of value to guiding behavior.

## Methods

### Participants

Data were collected from six healthy adult human subjects (four females, two males, mean age = 24, range = 21–26). All subjects had normal or corrected-to-normal vision. The study was approved by the University of California Berkeley committee for protection of human subjects. Written informed consent was obtained from all subjects prior to participation.

### Experimental stimuli

A total of 1620 stimuli were used, 1440 were presented during model estimation runs and 180 were presented during model validation runs. All stimuli were natural images obtained from the International Affective Picture System (IAPS) set^38^, the Lotus Hill image set^39^, and internet searches. The images were labeled using 23 mutually exclusive semantic categories: faces, full bodies, body parts, couples, gatherings (2–9 people), crowds (10+ people), land mammals, water mammals, fish, reptiles, insects, birds, savory food, dessert food, household items, vehicles, other artifacts, indoor buildings, outdoor buildings, land scenery, sky scenery, water scenery, plants. These semantic categories were based on those used by Naselaris and colleagues^26^ with a number of modifications (e.g. differentiation between reptiles and insects, elimination of irrelevant categories (namely texture patterns)). Four raters independently categorized each image; their modal categorization was used in the models described below. In just one instance no modal semantic category was obtained. This was resolved by discussion. The small number of images labeled as plants (*n* = 36 images) or vehicles (*n* = 72 images) led to the possibility of empty feature vectors when stimuli were labeled using compound features for semantic category by valence by arousal (see Model Features below). Hence, for all except the first two subjects to complete the first estimation and validation fMRI session (subjects 1 and 3), these images were removed and replaced with images from the buildings categories. Images were displayed at a visual angle of 12 × 12 degrees.

### Procedure

Subjects completed six fMRI sessions. Four 9.5 min retinotopy scans (two with clockwise/counterclockwise wedges and two with expanding/contracting rings^28^) were completed within session 1. Each of the subsequent five sessions comprised performance of the main task. In each of these sessions, participants completed six model estimation scans of 7.5 min duration and four model validation scans of 6 min duration. A structural scan was also acquired at the beginning of each session.

All stimuli were back projected onto a translucent screen positioned in the bore of the magnet, visible via an angled mirror placed above the subject’s head. Subjects fixated on a central white cross. Images were presented for 1 s with a 3 s inter-stimulus-interval during which the fixation cross was presented against a gray background with luminance matched to the mean luminance of the images in the stimulus set. During each estimation scan, forty-eight images were presented twice in a pseudo-random order. Null trials (no image presented) were also included, occurring once every eight trials. During validation scans, nine images were each presented nine times using a Type-1, Index-1 sequence^40^ to control for order effects. While viewing the images, subjects performed one of two tasks. Four subjects (1, 3, 5, and 6) were asked to categorize the valence of each image as negative, neutral, or positive. To control for effects of task, two subjects (2, 4) performed an alternate semantic categorization task, categorizing each image as human, animal, object, food, or building/scene. For these two subjects, valence categorizations were obtained from the post-scan behavioral sessions (see below).

#### Post-scan behavioral task

After the six fMRI sessions were completed, each subject returned to our lab to complete an additional behavioral task. Each image viewed within the fMRI sessions was re-presented to subjects with the same gray background and degree of visual angle as used in the fMRI sessions. Subjects were instructed to first rate the valence of each image as negative, neutral, or positive. Labels above the image indicated which button to press for each valence. Following valence categorization, the text above the image changed to indicate that image content should be rated for arousal using a nine-point scale. At the beginning of the task, subjects were instructed that ‘positive’ referred to “something you would want to look at, have, or be close to”, while ‘negative’ referred to “something you wouldn’t want to look at, have or be close to”. Arousal was categorized on a continuum from not emotionally intense at all (1) to extremely emotionally intense (9). The task was self-paced and took between 3 and 6 sessions to complete, with each session lasting 1.5 h.

### fMRI data acquisition

FMRI data were collected on a 3T Siemens TIM Trio scanner at the UC Berkeley Brain Imaging Center using a 32-channel head coil. An echo-planar T2*-weighted imaging (EPI) sequence was used with a decreasing slice series, repetition time (TR) = 2.0 s, echo time (TE) = 34 ms, flip angle = 74, voxel size = 2.4 × 2.4 × 3.0 mm, inter-slice gap = 0.75 mm, matrix size = 98 × 98, field of view = 224 × 224 mm. We prescribed 25 axial slices to cover all of temporal and occipital cortices, and as much of frontal and parietal cortices as possible. The first 5 volumes of each scan were discarded to allow for T1 equilibration effects. Anatomical data were collected using a T1-weighted MP-RAGE sequence with 1 mm isotropic resolution. A separate T1 was acquired at the beginning of each scan session. Pulse oximetry and respiration data were collected during fMRI data acquisition, using a Biopac recording system (Biopac MP150 Data Acquisition Unit, Biopac UIM100C with Nonin 8600FO for pulse oximetry, and Biopac RSP100C with Biopac TSD221 for respiration). Eye tracking was performed using an Avotec camera and Arrington Viewpoint software.

### fMRI data preprocessing

FMRI data were preprocessed using Matlab version 8.0 (The MathWorks, Natick, MA) and SPM8 (Welcome Department of Imaging Neuroscience, London, UK). Blood oxygen level dependent (BOLD) images were first converted from DICOM to NIFTI format. Next, diagnostics were run on the BOLD time series from each scan. Following an approach similar to that adopted by Power and colleagues^41^ and Carp^42^, bad volumes (with unusually high changes in mean whole-brain signal intensity) were identified using the time-series diagnostic tool tsdiffana.m (http://imaging.mrc-cbu.cam.ac.uk/imaging/DataDiagnostics). Among other indices, this tool calculates the mean square difference of voxel-wise signal intensities between each volume (n) and the previous volume (n-1) and divides this by the mean signal across the whole volume averaged over the whole time-series. Volumes (both n and n-1) were rejected using an absolute cutoff (the recommended default of 10) as this handles differences between subjects in the noisiness of data better than a within-subject percentile cut off. In line with findings by Power and colleagues^41^, bad volumes tended to correspond to those with notable spikes in movement. Bad volumes were replaced by the average of the volumes on either side. Subsequent to this initial data-cleaning step, image realignment was conducted to correct for within run head movement and to align images between runs. This was then followed by slice time correction. An ‘image on/off’ nuisance variable accounting for variance due simply to image presence was regressed out of the preprocessed BOLD data. This was coded with a 1 for volumes with a stimulus present and 0 for volumes with no stimulus present and convolved with a 4 bin FIR function (FIR time bins: 2–4 s, 4–6 s, 6–8 s and 8–10 s post stimulus onset). Low-frequency drift in the BOLD data was identified using a Savitzky-Golay filter with a 120 s window and 3rd degree polynomials and this was subtracted from the signal. The mean response for each voxel within each run was then subtracted from the BOLD data to account for differences in BOLD signal values across runs. Data were neither normalized to common space nor spatially smoothed in order to retain maximal resolution for our voxel-wise modeling.

### fMRI data modeling

#### Models constructed

Each image viewed was labeled with a distinct set of features corresponding to the feature space for each of the following models:

1. The ‘Semantic Only’ model. The feature space for this model comprised the following mutually exclusive semantic categories: Human-Face, Human-Body, Human-Body-Part, Human-Couple, Human-Gathering, Human-Crowd, Land-Mammal, Water-Mammal, Bird, Fish, Reptile, Insect, Savory-Food, Dessert-Food, Household-Object, Other-Artifact, Indoor-Building, Outdoor-Building, Water-Scenery, Sky-Scenery, Land-Scenery. Two subjects (1 and 3) also viewed a small number of images belonging to two additional categories: Plants and Vehicles (see the Experimental Stimuli section). Each image was given a ‘1’ for the semantic category to which it was most often allocated, across raters, and a ‘0’ for all other categories.
2. The ‘Combined Semantic, Valence and Arousal’ (CSVA) model. Ratings from each subject were used to create subject-specific labels for each image for valence (negative, neutral or positive) and arousal (high or low). For subjects who did the valence categorization task in the scanner, each image was presented either twice (for images shown in model estimation runs) or nine times (for images shown in model validation runs). In both cases, the modal valence value was used. For cases in which there was no modal value (e.g. for images categorized once as negative and once as neutral), if one of the categorizations was neutral, the image was coded as neutral. All other images without modal responses (e.g. those with one negative and one positive categorization) were excluded from analysis (mean = 24 images excluded per subject). For the two subjects who did the semantic task within the scanner, post-scan valence categorizations were used. Image arousal level (high, low) was determined using a within-subject median split on the post-scan 9-point ratings of image arousal; images rated below the median were categorized as low-arousal, and those equal to or above the median were categorized as high-arousal. These labels were used to subdivide each semantic category by valence and by arousal. In this manner, new compound features were created, each of which had a binary (1/0) value for semantic category, valence, and arousal (e.g. Human-Body, neutral valence, low arousal = ‘1’). In addition to these semantic by valence by arousal features, additional binary (present = ’1’, absent = ‘0’) features that carried information about both semantic and emotional image content were also incorporated (e.g. mutilated, rotten). As these typically only applied to one or two semantic categories, the following 18 compound semantic-emotion (SE) features were created and included in the CSVA model: Mutilated humans, Mutilated animals, Rotten food, Threat directed towards the viewer by a human aggressor, Threat-directed towards the viewer by an animal aggressor, Threat directed away from the viewer by a human aggressor, Threat directed away from the viewer by an animal aggressor, Romantic couples portrait (i.e. face only), Romantic couples full bodies, Human babies, Animal babies, Human social interaction portrait, Human social interaction single human (e.g. person playing golf), Human social interaction couples, Human social interaction gatherings, Erotica portrait, Erotica single human, and Erotica couples.
3. The ‘Semantic with Valence by Arousal for Animate Stimuli’ model. This model included compound semantic by valence by arousal features (as described in the CSVA model above) for images belonging to animate semantic categories. Only semantic features were included for images belonging to inanimate semantic categories (as in the Semantic Model). In other words, information about image valence and arousal was only included for stimuli belonging to animate semantic categories.
4. The ‘Semantic with Valence by Arousal for Inanimate Stimuli’ model This model included compound semantic by valence by arousal features (as described in the CSVA model above) for images belonging to inanimate semantic categories. Only semantic features were included for images belonging to animate semantic categories (as in the Semantic Model). In other words, information about image valence and arousal was only included for stimuli belonging to inanimate semantic categories.
5. The ‘Valence by Arousal’ model. This model included six compound features indicating the valence (negative, positive, neutral) and arousal (high, low) values for each image. Here, as for the CSVA model, subject-specific ratings of valence and arousal were used to model each participant’s data. The semantic category of each image was not included in the model. This resulted in each image being assigned a single compound feature that was a combination of subject defined valence and arousal (e.g. negative, low arousal).
6. The ‘Gabor’ Model. This model was used to assess the representation of low-level image structure, specifically variation in luminance contrast across the image. Model features comprised the results of filtering each image, after it was grayscaled and zero meaned, with a set of 474 Gabor filters that spanned 4 orientations (0, 45, 90, and 135 degrees) and 5 spatial frequencies (1.5, 3, 6,12 and 24 cycles/image) across a square grid of spatial locations covering the image (500 × 500 px). The filters were spaced using a grid determined separately for filters at each spatial frequency such that adjacent Gabor filters were separated by 3 standard deviations of the spatial Gaussian envelope. Feature weights were z-scored across all images to normalize differences in energy magnitude due to Gabor filter size. Image processing was conducted using the STRFlab toolbox for Matlab (strflab.berkeley.edu).

#### Model estimation

Model estimation was performed using custom code written in Matlab. For each subject, a design matrix was created for each model with regressors that indicated the presence (1) or absence (0) of each of the model’s features for each stimulus. Each of these feature regressors was convolved with a finite impulse response (FIR) filter, resulting in 4 new regressors for each feature, each one representing a time delay of 2–4, 4–6, 6–8 and 8–10 s from stimulus onset, respectively. Taking the dot product of these regressors with a set of linear weights is functionally equivalent to convolution of the original feature with a linear temporal kernel that has nonzero entries for 2–4, 4–6, 6–8, and 8–10 s delays. Six movement parameters, as estimated by SPM 8, were also included in the model as nuisance regressors.

For each subject, fMRI data from the model estimation runs were concatenated. L2-penalized (ridge) linear least square regression was used to find feature regressor weights which mapped the model features onto the BOLD time-series for each voxel. L2-penalized regression requires specification of a hyper-parameter lambda, which determines the amount of penalization applied during feature weight estimation (i.e. how much the feature weights are shrunk towards a Gaussian distribution). A range of 10 lambda values logarithmically scaled from 10^−9^ to 10^5^ were tested. K-folds cross-validation was used to determine the optimal value of lambda. Specifically, for each value of lambda, each model was fit on 9/10ths of the estimation data by selecting 27 of the 30 estimation runs without replacement. Using the weights estimated, voxel-wise BOLD time-series were predicted for the remaining 1/10th of the data. This was repeated until all runs had been included once in the held-out data. Concatenating the 10 predicted sections of the data resulted in a predicted time-series for the entire estimation dataset for each voxel. This complete predicted time-series was correlated with the actual recorded BOLD time-series and the single lambda value which produced the highest mean correlation value across all voxels was selected. Although selecting lambda individually for each voxel would almost certainly result in higher model performance, we opted to use a single value across all voxels in order to keep feature weights on the same scale and allow for subsequent principal components analysis of feature weights across voxels (see below). The final feature weights used were created by re-estimating the weights across all the estimation data using the selected best lambda value. This fitting procedure was repeated for each model and each subject.

#### Model validation

Model validation was performed using custom code written in Matlab. For each subject, the voxel-wise prediction accuracy of each model was assessed using the concatenated BOLD time-series from the validation runs. Completely new images were viewed within these runs. As in model estimation, a design matrix was constructed for each model by creating regressors to indicate the presence or absence of each model feature for each of the images viewed during the validation scans. This design matrix was then convolved with four FIR bins (2–4 s, 4–6, 6–8 s and 8–10 s post stimulus onset). The feature weights obtained during model estimation were multiplied with the FIR-convolved validation design matrix to produce voxel-wise predicted BOLD time-series for the 6760 s of validation data. We correlated these predicted time-series with the observed validation BOLD time-series to obtain estimates of model prediction accuracy for each voxel, which provide a metric of model fit that controls for over-fitting. Permutation testing was used to determine the significance of this correlation for each voxel for each model (for more details, see the Statistical Analysis section).

We note that correlation values reported here are not scaled by each voxel’s noise ceiling, and hence are lower than those reported in papers where such scaling is used. Noise ceiling calculations require exact repetition of validation stimuli in the same order. This has the advantage of allowing a measurement of the amount of explainable variance, assuming that responses to the same stimuli in the same order are constant. Given potential issues of habituation across multiple presentations for emotional stimuli, we chose to minimize order effects by presenting images in a pseudo-randomized Type-1, Index-1 sequence^40^. A consequence of this is that we separately predict voxel-wise responses to each individual presentation of a given stimulus. Inevitably this is noisier than predicting averaged responses across several stimulus presentations leading to lower raw prediction values but similar power to detect prediction significance given the increased number of data points available without averaging.

#### Model comparison

We compared the voxel-wise prediction performance of the CSVA model to that of the Semantic only and Valence by Arousal models. Voxels whose activity were significantly predicted by any one of the three models were included in these comparisons. Correlations between predicted validation BOLD time-series and actual validation BOLD time-series were computed for each voxel for each model as described above. We then calculated the proportion of these correlations that were greater for the CSVA model compared with the Semantic Only and Valence by Arousal models. We used a bootstrap test (2-tailed) to determine whether this proportion was significant. Please see the Statistical Analysis section for more details. The same method, and cortical mask was used to compare the Semantic Only model against the Semantic with Valence by Arousal for Animate Stimuli (SVAA) and the Semantic with Valence by Arousal for Inanimate Stimuli (SVAI) models.

#### PCA of CSVA model feature weights

Principal components analysis (PCA) of CSVA model feature weights, across voxels, allows us to identify consistent patterns of co-tuning to image features, i.e. to identify image features to which voxels tend to show similar responses. We conducted a PCA of CSVA model feature weights across all voxels within OTC where the CSVA model fit significantly (as assessed by significant prediction of the BOLD time-series for the validation dataset) and outperformed the Semantic Only model. The weights from the 4–6 s and 6–8 s FIR bins were averaged together for each feature, as these time points correspond to the peak of the BOLD hemodynamic response function (HRF). Non-centered PCA was applied to these weights. The feature weights associated with vehicle and plant semantic categories were excluded as only 2 of the 6 subjects viewed stimuli from these categories. Both group-level and subject-wise PCA analyses were conducted.

For the group-level PCA, voxels were concatenated across subjects. Some of the structure in feature tuning across voxels may merely reflect co-variance between stimulus features. Hence, we sought to identify the top group PCs for which the amount of variance explained was more than achieved by consideration of stimulus features alone. To address this, we used a jackknifing procedure. This resulted in the retention of the top three PCs. (Please see the Statistical Analysis section for further details).

We next calculated the similarity between the top three group PCs and the top three PCs from each subject-wise PCA. This was achieved by using leave-one-out cross validation (LOOCV) to compare feature loadings for the top three PCs from group-level and subject-wise PCAs of CSVA model feature weights. We conducted PCA on a given participant’s data and extracted the top three PCs. We then correlated the PC loadings, across features, with those of the top three PCs obtained from a group level PCA conducted with the data from the given participant left out. Since the ordering of the PCs is by explained variance, the ordering of components at the group level may not always be the same as that at the single subject level. To ensure we were comparing single subject PCs to group PCs that captured similar dimensions, the top three single-subject PCs were re-ordered so that each single-subject PC was matched to the group PC with which it had the highest correlation. If there were conflicts (where the highest correlation for 2 or more single subject PCs was with the same group PC), we resolved them by selecting the single subject PC that was closest to the group PC in its ordering. This was done recursively where necessary.

#### Interpreting the top 3 group-level PCs

By correlating theoretically informative dimensions with each of the group PCs it is possible to investigate the aspects of stimulus content encoded by each PC. Each theoretical dimension of interest was formalized as a vector comprised of values for each of the CSVA model features. We used three theoretical dimensions to explore the representation of animacy. The first of these comprised a four-level scale of animacy^18^ with inanimate objects at the bottom of the 0–3 point scale, followed by invertebrates and non-mammalian vertebrates, then non-human mammals, with humans at the top of the scale. We also used a simpler binary animacy dimension where features indicating that the stimulus was animate were given a ‘1’ and features indicating that the stimulus was inanimate were given a ‘0’. The third dimension specifically coded for the presence (1) or absence (0) of humans. The remaining dimensions encoded the perceived valence (positive = +1; negative = −1, neutral = 0) and arousal (high = 1; low = 0) of stimuli from either animate or inanimate semantic categories. As an example, the fourth dimension encoded the perceived valence of all animate stimuli. Here, all CSVA compound features indicating an image was perceived as positively valenced and belonged to one of the 12 animate semantic categories were given a ‘1’, all CSVA compound features indicating an image was perceived as negatively valenced and belonged to one of the 12 animate semantic categories were given a ‘−1’, and all CSVA compound features indicating an image was perceived as neutrally valenced and belonged to one of the 12 animate semantic categories were given a ‘0’. To determine the valence for each of the 18 semantic-emotion (SE) compound features in the CSVA model, we took the mode of the valence categorizations for stimuli possessing that feature across subjects. Finally, inanimate stimuli were given the value that equated to the mean of animate stimulus values, in order to exclude their influence on the correlation with the PCs. In a parallel fashion, we also created dimensions that coded inanimate stimulus valence, animate stimulus arousal and inanimate stimulus arousal.

The vector for each theoretical dimension of interest was correlated with the CSVA feature loadings for each of the PCs from the group-level PCA on CSVA model feature weights, across OTC voxels. We used a bootstrap procedure to assess the significance of these correlations. Please see the Statistical Analysis section for further details.

#### Prediction of behavioral responses

We next sought to test whether OTC tuning to image semantic and affective features could predict behavioral responses associated with the stimuli presented. To investigate this, Amazon Mechanical Turk (AMT) workers were shown 25 potential behavioral responses and asked to select the response(s) that would be appropriate to take if confronted with the content of a given image from our stimulus set. The 25 behavioral responses included were informed by recent theoretical work^1,43^ and extended to fit our stimulus set. They were as follows: Bond with, Take Care of Young with, Nurture/Raise, Procreate with, Be Affectionate with, Have Sex with, Play with, Gain Social Support from, Gain Nutrition from, Gain Gustatory Satisfaction from, Relieve Thirst with, Use to Facilitate Activity, Take Shelter in, Warm Oneself with, Empathic Response to Suffering, Empathic Response to Joy, Prolong Looking at, Curtail Looking at, Retreat from, Defend Self from Aggressor, Defend Self from Unwanted Sexual Attention, Avoid Predation by Fleeing, Avoid Predation by Freezing, Avoid Disease by Not Touching, Avoid Toxins by Not Consuming. In total 49 workers allocated a total of 27,516 behavioral responses with a mean of 2 behavioral responses per worker per image, with 9 workers evaluating the behavioral responses suited to the content of each image. Four of the behavioral response options (Relieve Thirst with, Warm Oneself with, Defend Self from Unwanted Sexual Attention, Avoid Predation by Freezing) were rarely selected (less than 1% of total responses) and hence excluded from further analyses.

In order to compare models’ prediction of behavioral responses, we conducted additional PCAs on voxel feature weights for the Gabor model, the Semantic Only model and the Valence by Arousal model, respectively. We included all OTC voxels where the model in question significantly fit the validation BOLD time-series. For both the CSVA model and these additional comparison models, we projected the vector of feature values for each image into the PCA space of each model. This was achieved by calculating the inner product of the images’ feature vector with each of the PC loading vectors. This effectively captures the information about the image in question carried by each component of OTC tuning for a given model. For models incorporating affective features (the CSVA and Valence by Arousal models), we used the modal values of valence and arousal, across subjects, for each image. For the Valence by Arousal model, this procedure resulted in 6 PC scores per image, for the Semantic Only model, this procedure resulted in 21 PC scores per image. For the remaining models, we retained the top 21 PC scores per image to match the number of PCs in the Semantic Only model. In addition, we conducted PCA on CSVA model image features, across images, concatenating design matrices across subjects. We also projected the vector of feature values for each image into this PCA space, retaining the top 21 PC scores per image.

The final step entailed using these PC scores to predict the behavioral responses allocated to each image. This effectively allows us to determine how well the tuning captured by each model predicts behavioral responses to each image. We conducted a series of regression analyses using PCs from each of the PCAs described above. We varied the number of PCs entered as regressors; this number was increased in steps of 1 from 1 to *n* (*n* = 21 features except for the Valence by Arousal model, where *n* = 6 features), see Fig. 7B. In order to control for over-fitting we used leave-one-out cross-validation (LOOCV) to calculate the amount of variance explained in the behavioral responses (*R*^2^). Please see the Statistical Analysis section for further details.

### Visualization of fMRI results

#### Flatmap construction and ROI labeling

Cortical flatmap construction was conducted using PyCortex^44^. This python tool makes use of the Freesurfer image analysis suite for cortical reconstruction and volumetric segmentation (http://surfer.nmr.mgh.harvard.edu/). Following initial automatic segmentation, white matter and pial surface maps were hand edited to remove any remaining artifacts and the surface was regenerated. In order to flatten the cortical surface, five relaxation cuts were made into the surface of each hemisphere and the surface crossing the corpus callosum was removed. The calcarine sulcus cut was made at the horizontal meridian in V1 using retinotopic mapping data from Session 1 as a guide.

Early visual regions V1-V4 were defined using the retinotopic mapping data. In addition, a reduced semantic model with 8 categories (Faces, Bodies, Body Parts, Multiple People, Animals, Food, Objects, Scenes) was fit to the estimation data and used to identify the following functional landmarks on each subject’s flat map: RSC, Retrosplenial Complex; OPA, Occipital Place area; LO, Lateral Occipital cortex; pSTS, Posterior Superior Temporal Sulcus; EBA, Extrastriate Body Area; OFA, Occipital Face Area; FFA, Fusiform Face Area; PPA, Parahippocampal Place Area; ATFP, Anterior Temporal Face Patch. We also label the following sulci: IPS, Intraparietal Sulcus; STS, Superior Temporal Sulcus; ITS – Inferior Temporal Sulcus; CoS, Collateral Sulcus; Post-CS, Post Central Sulcus; CS, Central Sulcus; SF, Sylvian Fissure. Note, these ROIs are only for orientation of the viewer and were not used to constrain any of our analyses.

#### Display of PC scores on the cortical maps

A RGB color key was used to project voxel-wise PC scores for the top three group PCs onto individual subjects’ cortical maps (red=loading on PC1, green=loading on PC2, blue=loading on PC3). Voxel-wise PC scores were calculated as the product of CSVA model feature weights for a given voxel by feature loadings for each PC. PC scores were thresholded at 6 standard deviations above and below 0, with values beyond the threshold given the maximal (or minimal) value. Thus, a value of 0 for a given color channel is 6 s.d.’s below a PC score of 0, a value of 128 for a given color channel has a PC score of 0, and a value of 255 is 6 s.d.’s above a PC score of 0.

### Statistical analysis

#### Model prediction significance testing

We calculated voxel-wise prediction accuracies for each model for each subject. As described in the Methods section above, the feature weights obtained during model estimation were multiplied with the FIR-convolved validation design matrix to produce voxel-wise predicted BOLD time-series for the validation data-set. We calculated voxel-wise prediction accuracy scores by correlating these predicted time-series with the observed validation BOLD time-series (*n* = 3380 time points per voxel). Permutation testing was used to determine the significance of model prediction for each voxel. The following procedure was used. We randomly shuffled validation data-set images without replacement, and convolved the resulting feature regressors with the FIR filters to create a randomized FIR-convolved validation design matrix. This randomized design matrix was then multiplied by the feature weights for each voxel from model estimation. This created a predicted validation BOLD time-series for each voxel. This predicted time-series was correlated with the observed validation BOLD time-series for the same voxel. This was repeated 5000 times for each voxel, producing a null distribution of prediction accuracy scores (i.e. correlations) for each voxel. Significance (one-tailed) was determined on a voxel by voxel basis by evaluating the proportion of values in this null distribution that fell below the actual prediction accuracy score for that voxel for the model in question. We corrected for multiple comparisons by using the Benjamini-Hochberg procedure to apply a false discovery rate (FDR) correction across all cortical voxels within the subjects’ flat-map (*q* < 0.05).

#### Model comparison

We used a bootstrap procedure to estimate a distribution of prediction accuracy scores for each voxel for each model by resampling across the 20 validation runs with replacement, 1000 times. Pairs of models were compared by examining the relative proportion of voxels better predicted by one model than the other, for each of these 1000 iterations. We calculated a *z*-value from the mean and standard error across these 1000 ratio values, after subtracting 0.5 (chance level, both models perform equally). A *p*-value (2-tailed) indicating the extent to which one model was superior to the other, across voxels was computed from the resulting z-value.

#### Principal component significance testing

We conducted group-level and subject-wise PCAs of CSVA model feature weights, across voxels (see section above for details of voxel selection and time-bin selection.) For the group-level PCA, voxels were concatenated across subjects. In order to determine which group PCs explained significantly more variance than stimulus features alone we conducted a jackknife test. Thirty sets of voxel-wise feature weights were created by holding out each estimation run in turn and estimating the feature weights for each voxel using the remaining twenty-nine runs. PCA was conducted on each of the resultant thirty sets of feature weights. A standard one-tailed jackknife test was used to determine whether the amount of variance across voxels explained by the top PC from each of these jack-knifed PCAs was significantly greater than that explained by the top stimulus feature PC (i.e. the first component from a PCA conducted on the presence or absence of stimulus features across images). This procedure was repeated for subsequent PCs until the difference in variance explained by the PC from the group PCA of CSVA model feature weights, across voxels, and the corresponding stimulus PC was no longer significant. This procedure was used to determine the group PCs retained for further consideration.

PCA was conducted across voxels within four ROIs, namely OTC, non-EVC OTC, OFC, non-OFC Frontal, in addition to a whole-cortex mask. Table S3 gives the number of voxel included in the group PCA for each ROI, following voxel section procedures.

We used leave-one-out cross validation (LOOCV) to compare feature loadings for the top three PCs from group-level and subject-wise PCAs of CSVA model feature weights. Significance was calculated via a permutation test where, for each of the three PCs in turn, the PC loadings of the group-minus-subject-X PCs were shuffled relative to subject X’s PC loadings. This resulted in a null distribution, and the group-minus-subject-X against subject X correlation coefficient was compared with the 95th percentile value from the null distribution.

#### Relating PCs to hypothetical dimensions

Three PCs were retained from the group PCA of CSVA model feature weights (see section above). We investigated the aspects of stimulus content encoded by each of these PCs by correlating each of them with hypothetical dimensions of interest. The hypothetical dimensions were formalized as vectors comprised of values for each of the CSVA model features (see section above for further details). To assess the significance of correlations between the PCs and the hypothetical dimensions (correlated across features, *n* = 144 features) we created a bootstrapped distribution for each correlation using randomized sampling with replacement. Specifically, we resampled CSVA model features with replacement 5000 times and re-estimated the correlations between the group PCs and hypothetical dimensions across the sampled features. In this manner, we obtained a distribution of 5000 values for each correlation. This distribution was then used to conduct a one-tailed bootstrap test for significance and to obtain the 95% confidence interval. An alpha threshold of 0.05 was used to determine significance.

#### Predicting behavioral responses from OTC tuning

We investigated how well OTC tuning, as captured by the CSVA model, could predict behavioral response to the images viewed. We compared this prediction performance with that of three alternative models of OTC tuning and with that achieved using image features (as labeled by the CSVA model) as opposed to brain responses to these features. In each case, feature dimensionality was reduced using PCA.

PCA was conducted on feature weights for each of the four models of OTC tuning considered (see section above), across OTC voxels, and the inner product of each images’ feature vector with each of the PC loading vectors calculated. This gave a score for each image on each PC for each model. PC scores for each image were also obtained using PCA on the CSVA model image features themselves.

These PC scores were entered into regression analyses to predict behavioral responses for each image (see Methods). We used leave-one-out cross-validation (LOOCV) to calculate the amount of variance in behavioral responses explained. This form of cross-validation controls for over-fitting of the behavioral responses. For each model, and each behavioral response, each of the images (*n* = 1620 images) was left out one at a time. The PC scores for the other images were then used to predict the proportion of raters that allocated the behavioral response to the left out image. This was conducted in turn for all of the images. The amount of variance explained was summed across iterations, giving the total amount of variance in workers’ selection of each behavior that could be explained by a given set of PC scores. This was repeated across behaviors. This LOOCV *R*^2^ value was then scaled by the total explainable variance in behavioral mappings (this reflecting the consistency in behaviors selected for each image across MTurk workers.) Bootstrapping across images was used to determine confidence intervals for the scaled LOOCV *R*^2^. Specifically, across 1000 iterations, images were randomly sampled with replacement, and the above procedure repeated. The resultant 1000 scaled LOOCV *R*^2^ values were used to calculate confidence intervals which were used as the threshold of a one-tailed hypothesis test of LOOCV *R*^2^ values.

#### Transforming prediction accuracies to *z*-values

We transformed model prediction accuracies to *z*-values for display on each subject’s cortical map (Fig. 2 and Figs. S2, S4–S7). To do so, we first converted the correlation of the predicted and actual validation BOLD time-series to a *t*-statistic using the following equation:

1 $${{{{{\bf{t}}}}}}={{{{{\bf{r}}}}}}*{{{{{\rm{sqrt}}}}}}(({{{{{\rm{n}}}}}}-2)/1-{{{{{{\bf{r}}}}}}}^{2})$$

where *r* is the Pearson correlation coefficient and *n* is the number of validation volumes (3380). We then converted that *t*-statistic to a *z*-value by first finding the probability of the *t*-value using the student’s-*t* cumulative distribution function, and then using the normal probability density function to find the *z*-value associated with that probability value.

### Reporting summary

Further information on research design is available in the Nature Portfolio Reporting Summary linked to this article.

### Supplementary information

Supplementary Information

Reporting Summary

### Source data

Source Data

## Data Availability

The fmri data for all subjects is available together with the CSVA model feature matrices via the Open Science Framework database - https://osf.io/b5pxu/. For data privacy reasons, structural images have been skull stripped prior to sharing. Source data are provided with this paper.

## Code availability

Voxel-wise modeling analysis code, with tutorials, is available at https://gallantlab.org/voxelwise_tutorials/voxelwise_modeling.html. Detailed specification of our modeling choices can be found within the README file provided in the OSF directory: https://osf.io/b5pxu/.
