# Supplementary material for: Occipital-temporal cortical tuning to semantic and affective features of natural images predicts associated behavioral responses
Source: Nat Commun. 2024 Jul 9;15:5531. doi: 10.1038/s41467-024-49073-8 (PMC11233618; doi:10.1038/s41467-024-49073-8)
Supplement: Supplementary file 1 — Supplementary Information [file 41467_2024_49073_MOESM1_ESM.pdf]

Supplementary Information

Title: Occipital-temporal cortical tuning to semantic and affective features of natural images predicts associated behavioral responses.

Index

Supplementary Methods:

Variance Partitioning

Controlling for physiological noise

Supplementary PCAs

Representational Similarity Analyses (RSA)

Supplementary References

Supplementary Tables S1-S4

Supplementary Figures S1-S20

Supplementary Methods

Variance Partitioning

Variance partitioning, also known as commonality analysis [S1], allows for the variance in BOLD data (across time points for a given voxel) explained by the CSVA model to be partitioned into unique variance terms for subsets of features and combinations of these sub-sets [S1,S2]. As illustrated in figure S6, we used variance partitioning to examine variance explained by the CSVA model over and above that attributable to semantic category or affective features alone.

We first fit models for the following subsets of features and combination of subsets: semantic category features (Sem, n=21 features), affective features (Aff: valence x arousal, n=6 features), semantic category features + affective features (Sem+Aff, n=27 features), semantic category x affective features (Sem.Aff, n=126 features), compound features that carry both semantic and emotional information, e.g. rotten food, mutilated humans, (SE, n=18 features), semantic category and SE features (Sem+SE, n=39 features), affective and SE features (Aff+SE, n=24 features), semantic category and affective and SE features (Sem+Aff+SE), semantic category x affective features + SE Features (Sem.Aff+SE, n=144 features). Note, for subjects 1 and 3, due to the additional semantic categories of plants and vehicles, there were 23 Semantic features (Sem), 138 semantic category x affective features (Sem.Aff) and 156 Sem.Aff +SE features; see main methods for futher details.

For each model, the ridge coefficient (lambda) was allowed to vary across voxels to allow for an accurate estimation of the optimal lambda, and thus highest explained variance, per voxel. Cross-validation (50-fold) was used to select the optimal lambda from a 20 value log-space ranging from 0-1000. The squared prediction accuracy (r^2) from these 9 models was then combined to determine the variance partitions as follows:

1. $\boldsymbol{\sigma^2_{U(Sem)} = r^2_{(Sem+Aff+SE)} - r^2_{(Aff+SE)}}$

2. $\sigma^2_{U(Aff)} = r^2_{(Sem+Aff+SE)} - r^2_{(Sem+SE)}$

3. $\sigma^2_{U(SE)} = r^2_{(Sem.Aff+SE)} - r^2_{(Sem.Aff)}$

4. $\sigma^2_{U(Sem.Aff)} = r^2_{(Sem.Aff+SE)} - r^2_{(Sem+Aff+SE)}$

5. $\sigma^2_{C(Sem+Aff)} = r^2_{(Sem+SE)} + r^2_{(Aff+SE)} - r^2_{(Sem+Aff+SE)} - r^2_{(SE)}$

6. $\sigma^2_{C(Sem+SE)} = r^2_{(Sem+Aff)} + r^2_{(Aff+SE)} - r^2_{(Sem+Aff+SE)} - r^2_{(Aff)}$

7. $\sigma^2_{C(Aff+SE)} = r^2_{(Sem+Aff)} + r^2_{(Sem+SE)} - r^2_{(Sem+Aff+SE)} - r^2_{(Sem)}$

8. $\sigma^2_{C(Sem+Aff+SE)} = r^2_{(Sem+Aff+SE)} + r^2_{(Sem)} + r^2_{(Aff)} + r^2_{(SE)} - r^2_{(Sem+Aff)} - r^2_{(Sem+SE)} - r^2_{(Aff+SE)}$

9. $\sigma^2_{C(Sem.Aff+SE)} = r^2_{(Sem.Aff+SE)} + r^2_{(Sem.Aff)} - r^2_{(Sem+Aff+SE)} - r^2_{(Sem+Aff)}$

Note: unique variance terms are denoted $\sigma^2_{C(XX)}$ for feature set XX, combined variance terms are denoted $\sigma^2_{C(XX+YY)}$ for combined variance between feature sets XX and YY.

Traditionally variance partitioning uses in-sample r^2, meaning the explained variance is estimated on the same data used to fit the models [S1]. Voxel-wise modeling assesses model fit using out-of-sample correlation, or prediction accuracy (r). Conducting variance partitioning on out-of-sample data introduces additional technical challenges. When partitioning in-sample explained variance, the sum of all the unique and shared terms is guaranteed to equal the variance explained by the full model. However, when partitioning out-of-sample data it is possible that the sum of the unique and shared terms are greater than the explained variance of the full model. To address this issue, we utilized a technique developed by de Heer and colleagues[S2] to estimate, and apply, a bias term per variance partition to account for over-fitting.

We estimated variance explained only by semantic category x affective interactions or addition of SE features (i.e. variance explained by mutilated land-mammals not land-mammals in general) by using the following feature sub-sets: $\sigma^2_{U(Sem.Aff)} + \sigma^2_{U(SE)} + \sigma^2_{C(Sem.Aff+SE)}$, i.e. partitions 3+4+9, see Fig S6. Put another way, this is the variance explained by the CSVA model minus

variance that could be explained by responses to either semantic category or affective features alone.

Controlling for physiological noise

Respiration and pulse-oximetry signals were obtained during fMRI data collection using a Biopac recording system (Biopac MP150 Data Acquisition Unit, Biopac UIM100C with Nonin 8600FO) for pulse oximetry, and Biopac RSP100C with Biopac TSD221 for respiration. Preprocessing was conducted using the Physiological Log Extraction for Modeling (PhLEM) Matlab Toolbox[S3]. This toolbox implements the RETROICOR method [S4] for creating nuisance regressors. A sine and cosine phase time-series are generated from the respiration data, and two cosine and sine phase time-series from the pulse-oximetry data, giving 6 time-series in total. The variance in the estimation and validation BOLD data explained by these nuisance regressors was removed. The CSVA model was fit to the residual data from the estimation runs and CSVA model feature weights obtained. Prediction accuracies were calculated using the residual validation data. Scatter plots were used to compare prediction accuracies with physiological noise controlled for in this fashion against those without physiological noise controlled for (as described in the main text). These are presented in Figure S3.

Supplementary PCAs

Using physiological noise controlled data.

PCA was conducted on OTC voxel-wise CSVA feature weights estimated using the physiological noise controlled data. The same voxels were used as for the PCA reported in the main text. Pearson correlation coefficients were estimated between the top 3 original group PCs

and those obtained using the physiological noise controlled data. Correlations between the top 3 “physio-removed’ CSVA PCs and the original CSVA PCs were extremely high (rs>0.99), indicating very little effect of physiological noise on the dimensionality of tuning within OTC .

Changing voxel selection criteria.

Non-EVC OTC. Retinotopic mapping (using both rotating wedges and contracting and expanding circles) was used to define early visual cortex (EVC, namely V1-V4) for each participant. Voxel section criteria was as for the main OTC analysis described above with the additional criteria that only voxels in non-EVC OTC were included. See Figure S9 and Table S3.

Whole-cortex (expanded selection).

Here we included all cortical voxels where the CSVA model showed a significant fit (see Table S3). Figure S8A shows the amount of variance explained by the top 10 group-level PCs of this expanded-voxel-selection PCA, and figure S8B shows the correlation between the top three PCs from this analysis and those from the OTC-restricted PCA reported in the main text. Figure S11 shows the projection of PC scores from this supplementary PCA onto cortical flatmaps.

Orbital frontal cortex (OFC).

A structural OFC ROI was created by combining superior, middle, inferior and medial OFC ROIs from the AAL template [S5]. The resultant ROI was back-projected from MNI space to subject anatomical space using a non-linear transformation (spatial normalization from SPM8), and then into subject functional space using a linear 12-dimensional affine transformation (spatial co-registration from SPM8).

All voxels within this ROI where the CSVA model showed a significant fit were included in a PCA of CSVA model feature weights (see Table S3). Figure S12A shows the amount of variance explained by the top 10 group-level PCs. We used leave-one-out cross validation (LOOCV) to compare feature loadings for the top three group-level PCs and the top three PCs obtained by subject-wise PCAs (see Figure S12C). Figure S13 shows the projection of PC scores from this PCA onto cortical flatmaps.

Non-OFC frontal cortex.

This ROI was created by combining the following 5 AAL template structural ROIs: superior, middle, and superior medial frontal regions, as well as frontal inferior operculum and frontal inferior triangularis. These ROIs were back-projected into subject functional space in the same manner as for the OFC ROI.

All voxels within this ROI where the CSVA model showed a significant fit (see Table S3) were included in a PCA of CSVA model feature weights. Figure S12B shows the amount of variance explained by the top 10 group-level PCs. We used leave-one-out cross validation (LOOCV) to compare feature loadings for the top three group-level PCs and the top three PCs obtained by subject-wise PCAs (see Figure S12D). Figure S14 shows the projection of PC scores from this PCA onto cortical flatmaps.

PCA controlling for low-level image features

To determine the extent to which tuning to CSVA model features is merely explained by covariance with low level image features, we fit the Gabor model to the estimation data and regressed out the variance explained before fitting the CSVA model to the residuals. PCA was

conducted on the resultant CSVA model feature weights for OTC voxels divided into (a) non-EVC OTC and (b) EVC. See Figure 7.

Representational Similarity Analyses (RSA)

Prior work using multivoxel pattern analysis (MVPA) and specifically Representational Similarity Analysis (RSA) has failed to find interactions of stimulus valence and animacy in OTC [S6]. This might reflect the smaller amount of fMRI data per subject used in this prior work resulting in lower within-subject power to detect effects. Alternatively, RSA based MVPA might be less sensitive than the voxel-wise modeling approach adopted here, especially for studying voxel response profiles in a multi-dimensional space. This issue is likely to be of interest to some readers. Hence we conducted supplementary analyses using MVPA based RSA to relate image similarity in a space defined by image animacy, arousal and valence (see Table S5) to similarity in activation patterns across OTC. We conducted additional parallel analyses for OTC excluding EVC and for the two frontal ROIs (OFC and non-OFC frontal cortex). We also conducted RSA using semantic only and Valence by Arousal model (dis)similarity matrices (see Table S5).

Methods

Representational similarity analysis (RSA) is conducted by correlating representational dissimilarity matrices which quantify (dis)similarity, across images, in BOLD activity patterns (brain RDMs) with model-based RDMs which quantify (dis)similarity, across images, in model feature space.

We first preprocessed the BOLD data using SPM 8, conducting slice time correction, realignment, and linear detrending. High-pass filtering was not used (beyond linear detrending)

to avoid the accidental removal of low-frequency stimulus signals resulting from only 2 presentations of each stimulus. Spatial smoothing was also not conducted [S7].

SPM 8 was then used to estimate a t-contrast across all brain voxels for each of the 1440 estimation stimuli, which quantified each voxel's average response to each stimulus relative to baseline. Nuisance regressors accounting for movement were included in the model, as well as a constant bias term per run to account for differences in baseline BOLD activity between runs. Each unique image was assigned an event, and the resulting t-contrast for that event was used to obtain the voxel-wise response amplitudes for the brain RDM. Implicit masking was disabled. Explicit masks were used to create ROIs using the same voxel-selection criteria as adopted for our voxel-wise modeling analyses. ROIs comprised OTC (as in main manuscript), non-EVC OTC, OFC and non-OFC frontal cortex. For each of these ROIs, a single brain RDM was estimated using pairwise Pearson's correlations between the response amplitudes (t-contrast values) of all selected ROI voxels for each of the 1440 estimation stimuli (i.e. so the response to stimulus one was correlated with that to stimulus two, across voxels and so on). To create a group averaged brain RDM, single subject RDM values were Fisher transformed, averaged across subjects, and then reverse Fisher transformed for visualization. In addition to conducting formal RSA analyses (see below), we divided stimuli as a function of stimulus conditions and plotted group-level dissimilarity matrices for the following ROIs: EVC (early visual cortex), non-EVC OTC, OFC, non-OFC frontal cortex, and across the whole brain (Figure S18.)

Each of the nine model RDMs (as described in Table S5 below) was created by estimating a distance metric for each of the 1440 estimation images within the model feature spaces outlined in Table S5. This was conducted separately for each subject using their individual ratings of valence and arousal. RSA was conducted using the CosMoMVPA toolbox[S8]. We estimated

Spearman rank-order correlations between each ROI brain RDM and each model RDM (Fig S19). We also conducted partial spearman correlations to control for variance attributable to low-level visual features (using a Gabor model RDM).

Single subject RSA results were Fisher transformed, averaged (across subjects), and reverse Fisher transformed for visualization of group results. To calculate significance of these group RSA results, the subject-specific correlation values were Fisher transformed and subjected to one-tailed t-tests across subjects. These results were corrected for multiple comparisons across ROIs and models (using Bonferroni correction).

**Model-based RDMs**	**Distance Metric**	**Values**
Animacy 4 level	Euclidean in 1-D	1 unit distance between adjacent cells, ordered as follows: Human, non-human Mammal, Invertebrate, or Inanimate
Animacy 2 level	Binary	0 distance if in same cell, 1 unit distance if not: Animate, Inanimate.
Semantics	Binary	21 semantic categories from the CSVA model; 0 distance if in same category, 1 unit distance if not:
Valence	Euclidean in 1-D	1 unit distance between adjacent cells, ordered as follows: Negative, Neutral, or Positive.
Arousal	Binary	High Arousal or Low Arousal; 0 distance if in same cell, 1 unit distance if not.
Valence by Arousal	Euclidean in 2-D	6 points in a 2-D space: high-negative, low-negative, high-neutral, low-neutral, high-positive, and low-positive. Space definition: distance of 1 between each adjacent valence cell and distance of 1 between arousal levels.
Animacy by Valence by Arousal	Euclidean in 3-D	The 6 points above are replicated for both animate and inanimate cells. Giving a 3-D space. Distance of 1 between matching animate and inanimate cells.
Animacy by Valence	Euclidean in 2-D	6 points 2-D space: animate-negative, animate-neutral, animate-positive, inanimate-negative, inanimate-neutral, and inanimate-positive. Distance of 1 between each adjacent valence cell and distance of 1 between animate and inanimate cells of the same valence.
Animacy by Arousal	Euclidean in 2-D	4 points 2-D space: animate-high, animate-low, inanimate-high, and inanimate-low. Distance of 1 between arousal levels (same animacy) and distance of 1 between animacy levels (same arousal.)

Table S5. Description of the model-based representational (dis) similarity matrices. The left-most column describes the feature space implemented; the middle column describes the distance metric used and the right-most column describes matrix cells and distances. We include this table here, as opposed to at the end of the Supplements, for the reader’s convenience. Each model-based RDM was fit at a single subject level using the individual subject’s ratings of valence and arousal, as described for the CSVA and Valence by Arousal encoding models.

Results.

One of the limitations with RSA is that arbitrary decisions need to be made about the distances between features. This is particularly an issue when using 2D or 3D feature spaces to examine interactions. The assumptions we have made are spelt out in Table S5. We note that the voxel-wise encoding modeling approach benefits from not requiring any assumptions about distances between features. With this caveat noted, we report that RSA revealed representation of interactions between animacy, valence and arousal in both OTC and OTC with EVC excluded. This representation was less evident in frontal regions (Figure S19). Controlling for similarity in low-level image structure did not noticeably alter these results (Figure S19). If we replace our OTC ROI with a ventro-temporal cortical ROI used elsewhere[S6], representation of interactions between animacy, valence and arousal are still observed (Figure S20). Inspection of the brain RDMs with stimuli collapsed by stimulus category (Figure S18) allows for post-hoc detection of patterns that might have been missed due to assumptions operationalized when deciding upon feature distances for the model RDMs. Across all ROIs, and the whole brain, an animate vs. inanimate divide can be seen, although it is much more prominent within non-EVC OTC, as would be expected from previous findings. Stimuli are more differentiated by affective properties within non-EVC OTC than in other regions but some differentiation by affective properties is still discernable within frontal regions (e.g. neutral low arousal inanimate stimuli show the highest dissimilarity to positive and negative high arousal animate stimuli).

Supplementary References

1. Nimon, K., & Reio, T. G. Regression commonality analysis: A technique for quantitative theory building. *Human Resource Development Review*, **10**(3), 329–340 (2011).
2. De Heer, W. A., Huth, A. G., Griffiths, T. L., Gallant, J. L., & Theunissen, F. E. The Hierarchical Cortical Organization of Human Speech Processing. *The Journal of Neuroscience*, **37**(27), 6539–6557 (2017).
3. Verstynen, T. D. & Deshpande, V. Using pulse oximetry to account for high and low frequency physiological artifacts in the BOLD signal. *NeuroImage*, **55**(4), 1633–1644 (2011).
4. Glover, G. H., Li, T. Q. & Ress, D. Image-based method for retrospective correction of physiological motion effects in fMRI: RETROICOR. *Magnetic Resonance in Medicine*, **44**, 162–167 (2000).
5. Tzourio-Mazoyer, N. et al. Automated anatomical labeling of activations in SPM using a macroscopic anatomical parcellation of the MNI MRI single-subject brain. *NeuroImage*, **15**, 273–289 (2002).
6. Chikazoe, J., Lee, D. H., Kriegeskorte, N., & Anderson, A. K. Population coding of affect across stimuli, modalities and individuals. *Nat. Neurosci.* **17**, 1114-1122 (2014).
7. Diedrichsen, J. & Kriegeskorte, N. Representational models: A common framework for understanding encoding, pattern-component, and representational-similarity analysis. *PLoS Comput Biol*. **13**(4):e1005508 (2017).
8. Oosterhof, N. N., Connolly, A. C., & Haxby, J. V. CoSMoMVPA: Multi-Modal Multivariate Pattern Analysis of Neuroimaging Data in Matlab/GNU Octave. *Frontiers in Neuroinformatics* **10**, 1–27 (2016).

Supplemental Tables

Subject	# Voxels used in Model Comparison
1	6777
2	6450
3	5483
4	6596
5	6580
6	6618

Table S1. This table shows the number of voxels used for all model comparison analyses (see the main methods section for details of model comparison procedures).Voxel selection was done by selecting those voxels where one of the following three models shows a significant fit: CSVA, Valence by Arousal, or Semantic Only.

**Subject**	**Component 1**		**Component 2**		**Component 3**	
	**r-value**	**p-value**	**r-value**	**p-value**	**r-value**	**p-value**
1	0.899	*P=1E-8*	0.867	*P=1E-8*	0.811	*P=1E-8*
2	0.904	*P=1E-8*	0.707	*P=1E-8*	0.722	*P=1E-8*
3	0.825	*P=1E-8*	0.655	*P=1E-8*	0.780	*P=1E-8*
4	0.901	*P=1E-8*	0.455	*P=1E-8*	0.497	*P=1E-8*
5	0.781	*P=1E-8*	0.772	*P=1E-8*	0.544	*P=1E-8*
6	0.206	0.0065	0.674	*P=1E-8*	0.662	*P=1E-8*

Table S2. Results from a leave-one-out cross validation analysis comparing PC feature loadings from subject-wise and group-level PCAs of CSVA feature weights across OTC voxels. For each of the top three principal components, the Pearson correlation coefficient (r-value) and associated p-value are given for the correlation between single subject PCA feature loadings and the feature loadings from a group PCA conducted with data from that given subject excluded. Permutation tests were used to determine correlation significance (one-tailed). Note a p-value of 1E-8 is the smallest possible value given the permutation test used.

**ROI Name**	**# Voxels included in group PCA**
OTC	8708
non-EVC OTC	7208
OFC	517
non-OFC Frontal Cortex	2517
Whole Cortex	26107

Table S3. This table gives the number of voxels (concatenated across participants) included in the group-level principal component analyses (PCAs). Voxel counts are given for the Occipital Temporal Cortical (OTC) ROI used in our primary analyses (see main manuscript) and the additional ROIs used for the supplementary PCAs described here. EVC = Early Visual Cortex; OFC = Orbital Frontal Cortex

Figure	Description	Source	License
1	Lion	https://stock.adobe.com/ie/images/lion-en-pleine-nature/284349908?prev_url=detail	Abobe stock extended licence
1	Prison	https://stock.adobe.com/ie/images/wide-angle-view-of-jail-or-prison-bars-being-lit-up-by-the-sun/318331805?prev_url=detail	Abobe stock extended licence
1	Woman on bench	Author's private photo	Provided by authors
1	Candles	http://christmasstockimages.com/free/ideas_concepts/slides/christmas_candles.htm	CC BY 3.0
1	Father & daughter	Author's private photo	Provided by authors
1	Flood under bridge	https://commons.wikimedia.org/wiki/File:Flood_under_the_Old_Route_49_bridge_crossing_over_the_South_Yuba_River_in_Nevada_City,_California.jpg	CC0 1.0
8	Baby's face	https://stock.adobe.com/images/cute-baby-ginger-hair-close-up-crawling-on-bed-smiling-adorable-kid-portrait-family-lifestyle-3-month-old-child/302722731?prev_url=detail	Abobe stock extended licence
8	Couple embracing	https://stock.adobe.com/images/content-young-african-couple-embracing-each-other-at-the-beach/183353446?prev_url=detail	Abobe stock extended licence
8	Villa	https://pxhere.com/en/photo/1196675	CC0 1.0
8	Beef fillet on plate	https://en.wikipedia.org/wiki/File:Beef_fillet_steak_with_mushrooms.jpg	CC BY-SA 3.0
8	Tools on wall	https://www.pexels.com/photo/set-of-tool-wrench-162553/	CC0 1.0
8	Tsunami	https://stock.adobe.com/images/raz-de-maree-sur-la-ville/2073696?prev_url=detail	Abobe stock standard licence
8	Man in hospital bed	https://stock.adobe.com/images/paramedic-checking-up-and-using-flashlight-on-injured-young-man/419848982?prev_url=detail	Abobe stock extended licence
8	Snarling dog	https://stock.adobe.com/images/an-angry-dog-barks-near-the-house/202435947?prev_url=detail	Abobe stock standard licence
8	Man vomiting in toilet	https://stock.adobe.com/ie/images/man-vomiting-in-the-toilet/109253754?prev_url=detail	Abobe stock extended licence

Table S4. This table provides the source URL and license type for all images shown within figures of this paper.

Supplemental Figures

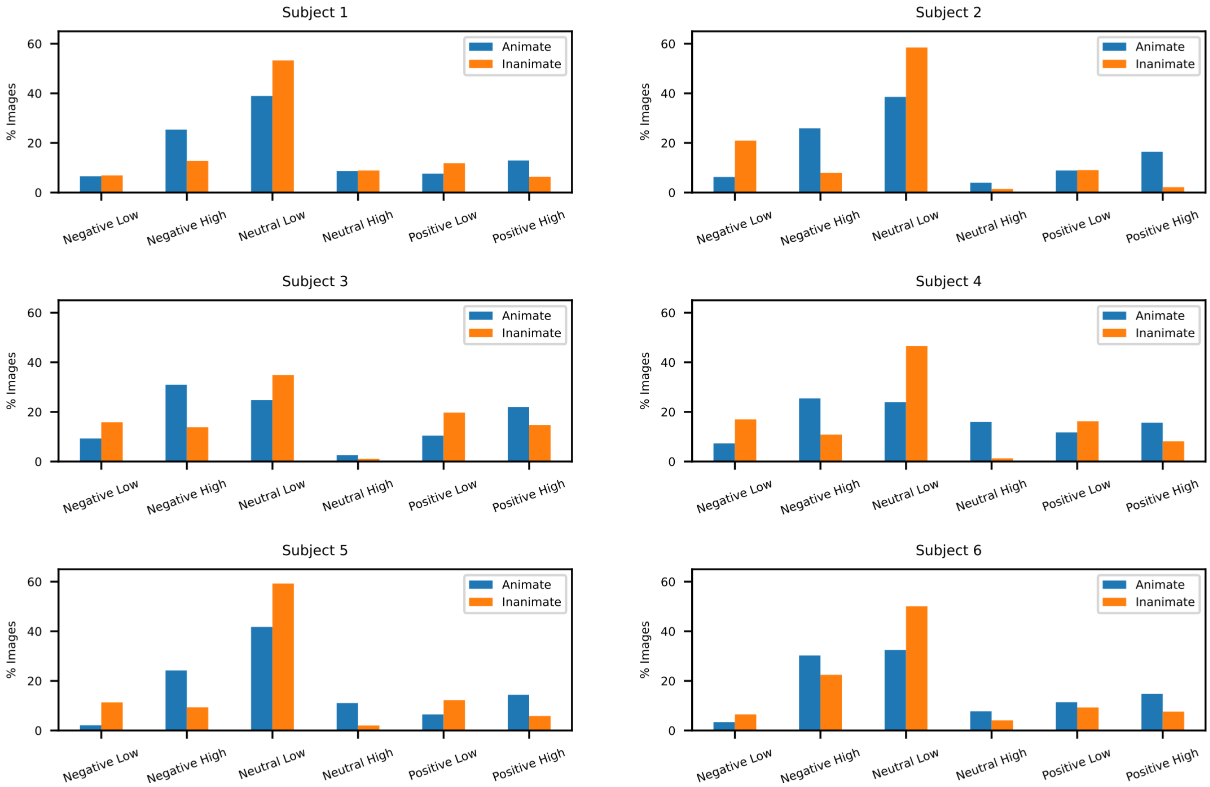

Figure S1 – Percentage of animate images (blue bars) and inanimate images (orange bars) classified as negative, neutral or positive and as high or low arousal for each subject. Ratings from each subject were used to create subject-specific labels for each image for valence (negative, neutral or positive) and arousal (high or low), see Methods. Image arousal level (high, low) was determined using a within-subject median split on the post-scan 9-point ratings of image arousal conducted across all images regardless of semantic category. Images rated below the median were categorized as low-arousal, and those equal to or above the median were categorized as high-arousal.

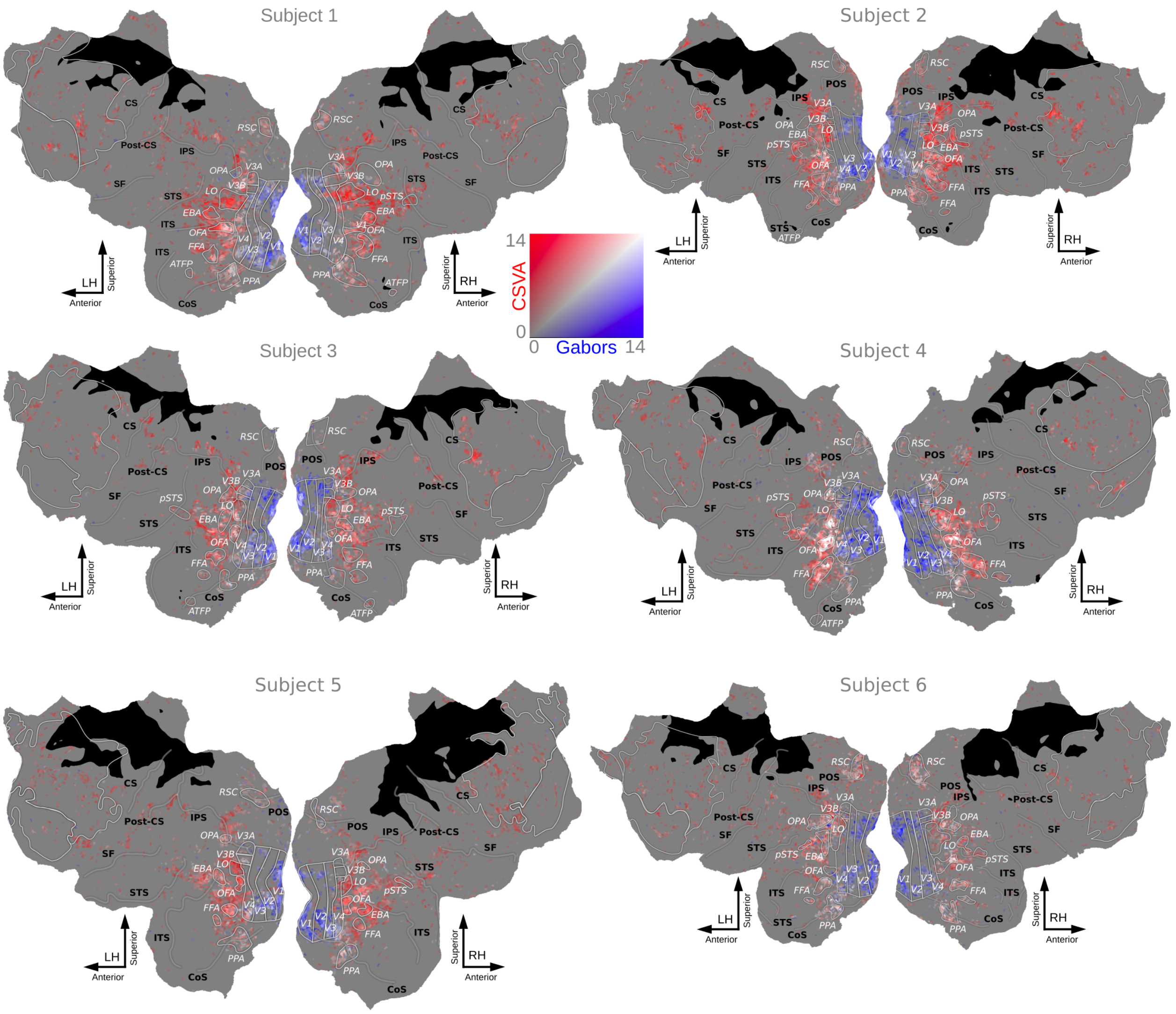

Figure S2. Cortical maps for each subject showing voxel-wise prediction accuracies for the Combined Semantic, Valence and Arousal (CSVA) model and the Gabor model. Voxel-wise prediction accuracy values for each model were z-transformed and coded using a two-dimensional color key with blue representing *Gabor* model prediction accuracy and red representing CSVA model prediction accuracy. For each channel, minimum color intensity corresponds to a z-transformed prediction accuracy of 0 and maximum color intensity corresponds to a z-transformed prediction accuracy of 14. In all subjects, the CSVA model shows superior performance to the Gabor Model in OTC regions outside of early visual cortex (V1-V4). As expected from prior work, the Gabor model performs well in V1-V4. White voxels are those where both models fit extremely well. Note: Regions of interest (ROIs) are labeled in white, sulci in black. RSC: Retrosplenial Complex, OPA: Occipital Place Area, LO: Lateral Occipital cortex, pSTS: Posterior Superior Temporal Sulcus, EBA: Extrastriate Body Area, OPA: Occipital Place Area, OFA: Occipital Face Area, FFA: Fusiform Face Area, PPA: Parahippocampal Place Area, ATFP: Anterior Temporal Face Patch. IPS: Intraparietal Sulcus, STS: Superior Temporal Sulcus, ITS: Inferior Temporal Sulcus, CoS: Collateral Sulcus, CS: Cental Sulcus, Post-CS: Postcentral Sulcus, SF: Sylvian Fissure, POS: Parieto-Occipital Sulcus

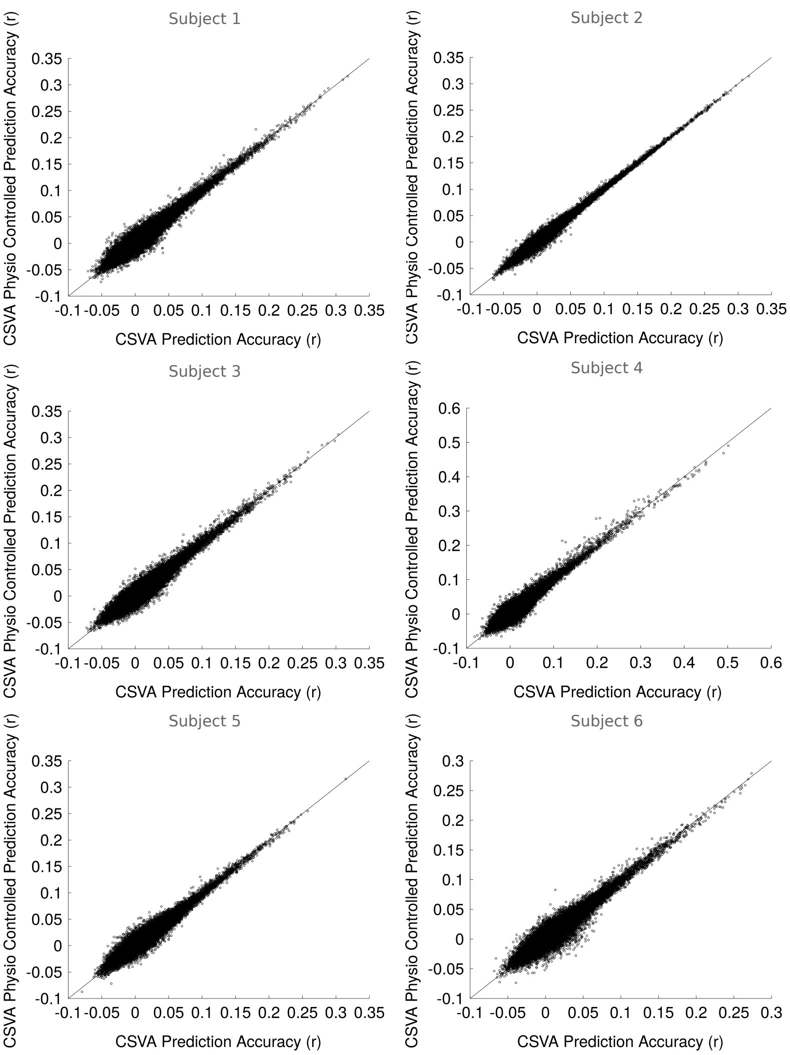

Figure S3. Similarity in Prediction Accuracy after Controlling for Physiological Noise. Respiration and pulse-oximetry measurements were obtained during fMRI data acquisition The PhLEM Matlab Toolbox was used to create nuisance regressors that capture low-frequency phase information contained in these measurements (see Supplementary Methods). Variance in the BOLD data explained by these nuisance regressors was estimated using linear regression. This variance was partialled out and the CSVA model refit to the residual BOLD timeseries. Scatter plots comparing the voxel-wise prediction accuracy values from the CSVA model and the CSVA Physio-Controlled model are shown here. Points on the x=y line show equal performance for both models; points above the line indicate voxels where the model on the y-axis (CSVA Physio Controlled) performed better, and points below the line indicate voxels where the model on the x-axis (CSVA) performed better. It can be seen that controlling for contributions of physiological noise to the BOLD signal has little systematic impact on CSVA model fit. We note that when PCA was performed on CSVA feature weights obtained using the physio-controlled data, feature loadings on the top three PCs were highly correlated with those obtained without physiological noise correction, rs >0.99.

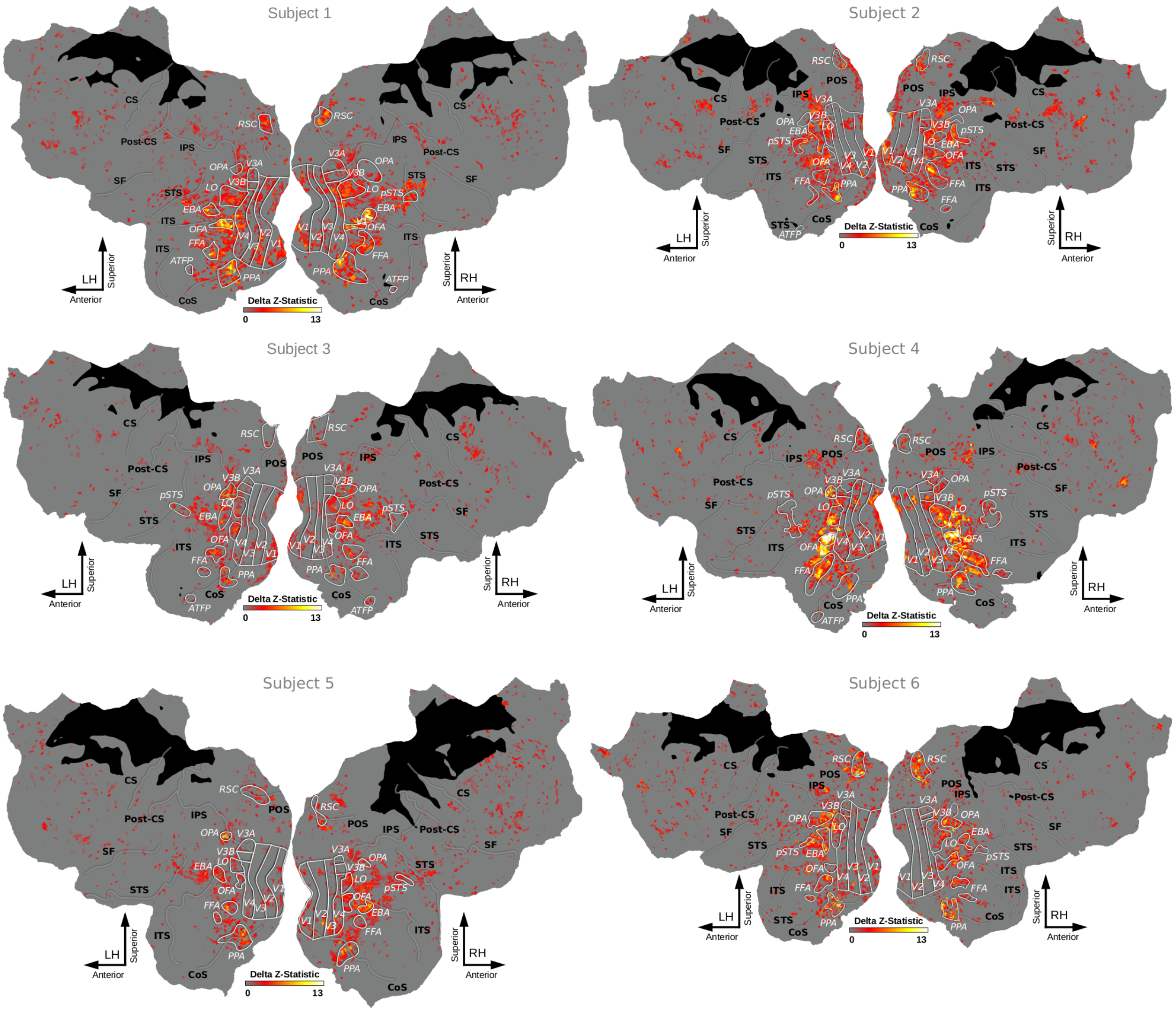

Figure S4. Improvement in voxel-wise prediction accuracies for the CSVA model relative to the Valence by Arousal Model.

Cortical maps for each subject show voxels where prediction accuracy was greater for the *CSVA* model than the *Valence by Arousal* model. Voxel-wise prediction accuracy values for each model were z-transformed and subtracted (see the Statistical Analysis section of the Methods for full details). Only voxels whose activity was significantly predicted by any one of the following three models were included in these comparisons: *CSVA*, *Valence by Arousal* and *Semantic Only*.

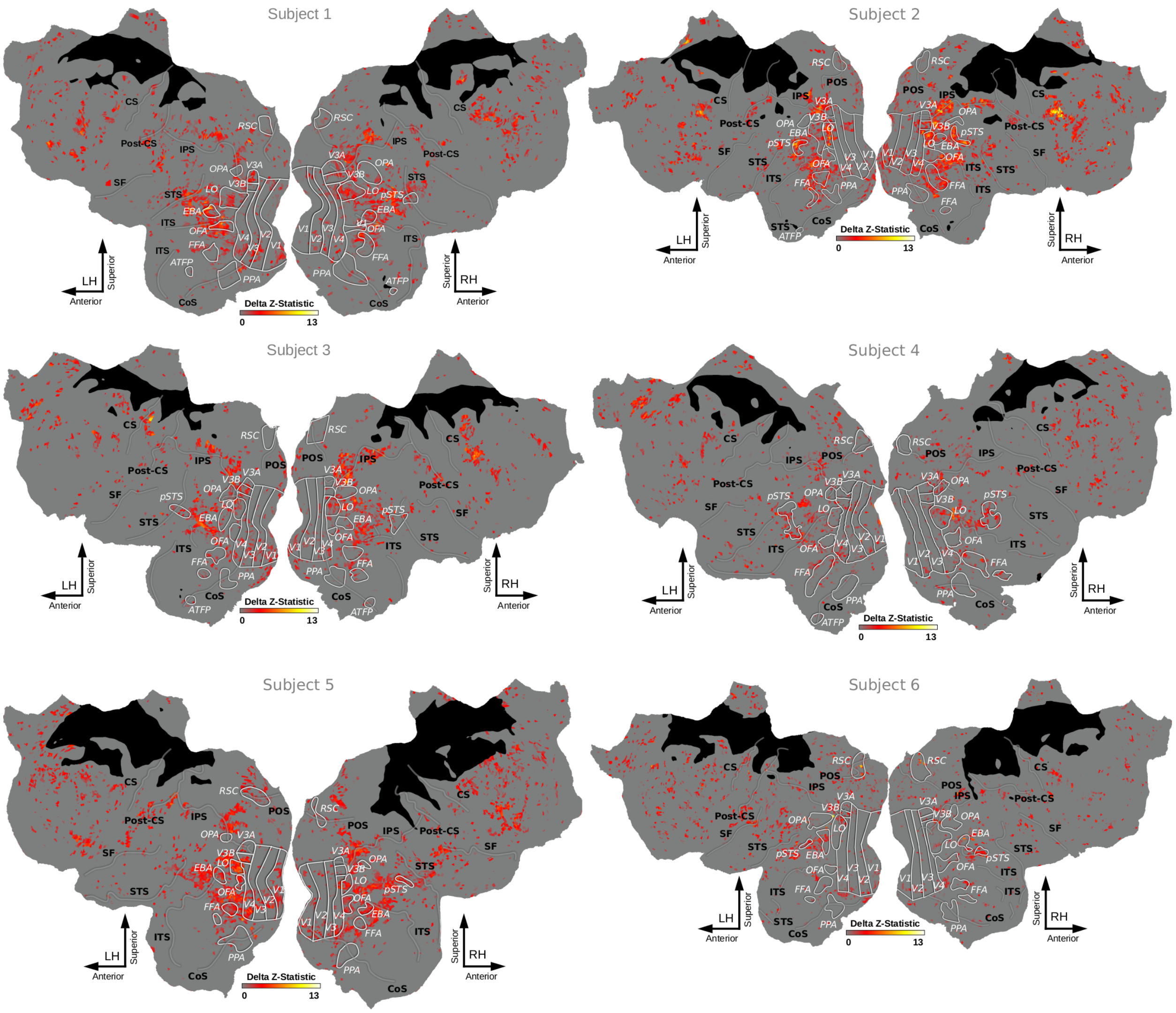

Figure S5. Improvement in voxel-wise prediction accuracies for the CSVA model relative to the Semantic Only Model.

Cortical maps for each subject show voxels where prediction accuracy was greater for the *CSVA* model than the *Semantic Only model*. Voxel-wise prediction accuracy values for each model were z-transformed and subtracted (see the Statistical Analysis section for full details). Only voxels whose activity was significantly predicted by any one of the following three models were included in these comparisons: *CSVA*, *Valence by Arousal* and *Semantic Only*.

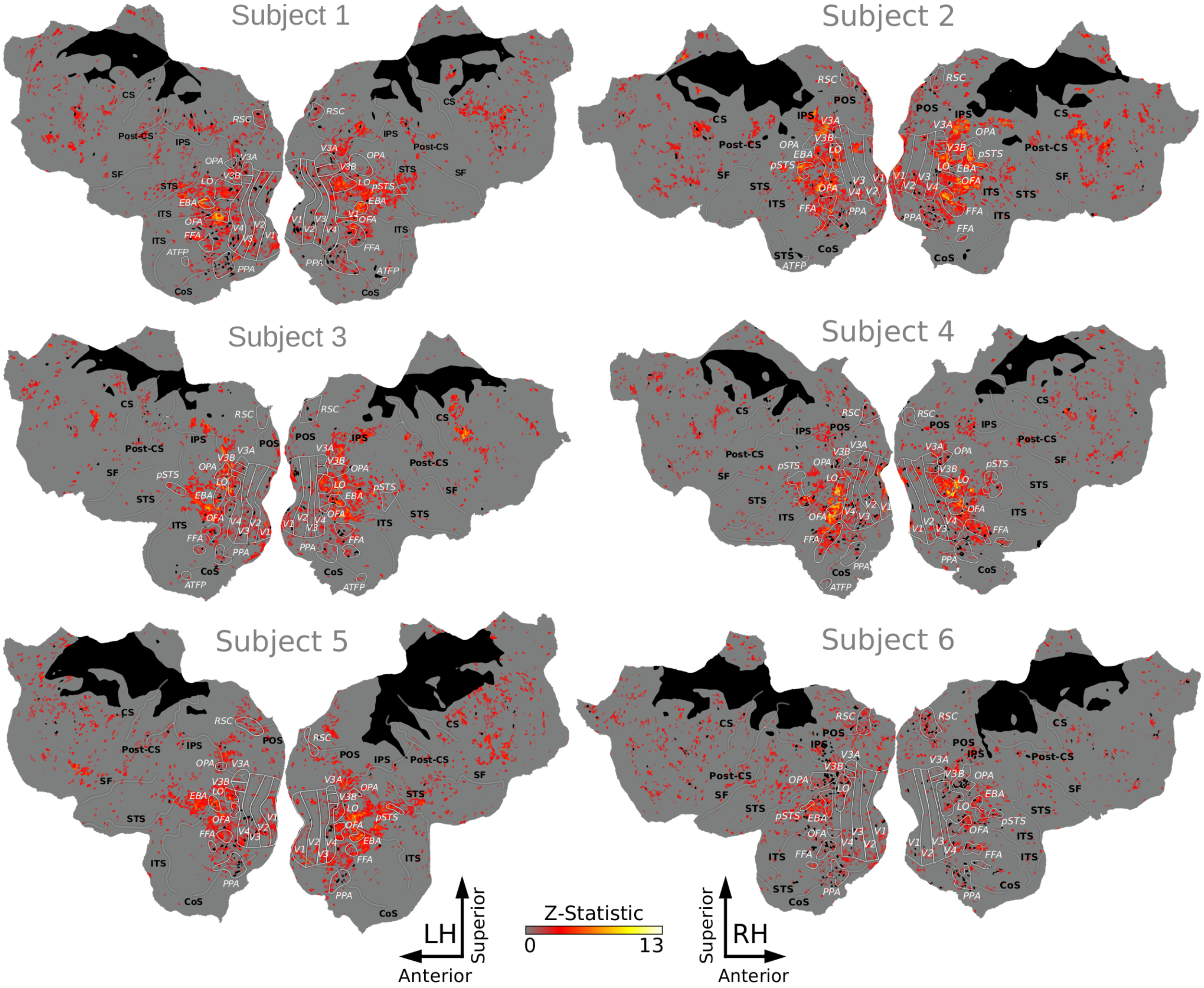

Figure S6. Variance partitioning results: individual maps showing voxel-wise responses uniquely attributable to semantic category by affective feature interactions and semantic-emotion (SE) compound features. Variance partitioning was used to remove variance reflecting responses to semantic category features alone or affective features alone while leaving variance uniquely attributable to semantic category by affective feature interactions and SE compound features. (To explain this through an example, variance in a voxel time-series attributable to the semantic category feature ‘land-mammals’ and to the affective feature ‘high-arousal negative images’ were effectively removed whereas variance unique to the semantic category x affective feature interaction ‘high arousal negative land-mammals’ and to the SE compound feature ‘mutilated land-mammals’ was retained; for actual partitioning methods see Supplementary Methods.) Voxel-wise residual prediction accuracies are shown for each subject. Many of the OTC voxels fit by the full CSVA model (see Figure 2 in the main manuscript) continue to show significant prediction accuracy scores. Note: Regions of interest (ROIs) are labeled in white, sulci in black. RSC: Retrosplenial Complex, OPA: Occipital Place Area, LO: Lateral Occipital cortex, pSTS: Posterior Superior Temporal Sulcus, EBA: Extrastriate Body Area, OFA: Occipital Face Area, FFA: Fusiform Face Area, PPA: Parahippocampal Place Area, ATFP: Anterior Temporal Face Patch. IPS: Intraparietal Sulcus, STS: Superior Temporal Sulcus, ITS: Inferior Temporal Sulcus, CoS: Collateral Sulcus CS: Cental Sulcus, Post-CS: Postcentral Sulcus, SF: Sylvian Fissure, POS: Parieto-Occipital Sulcus

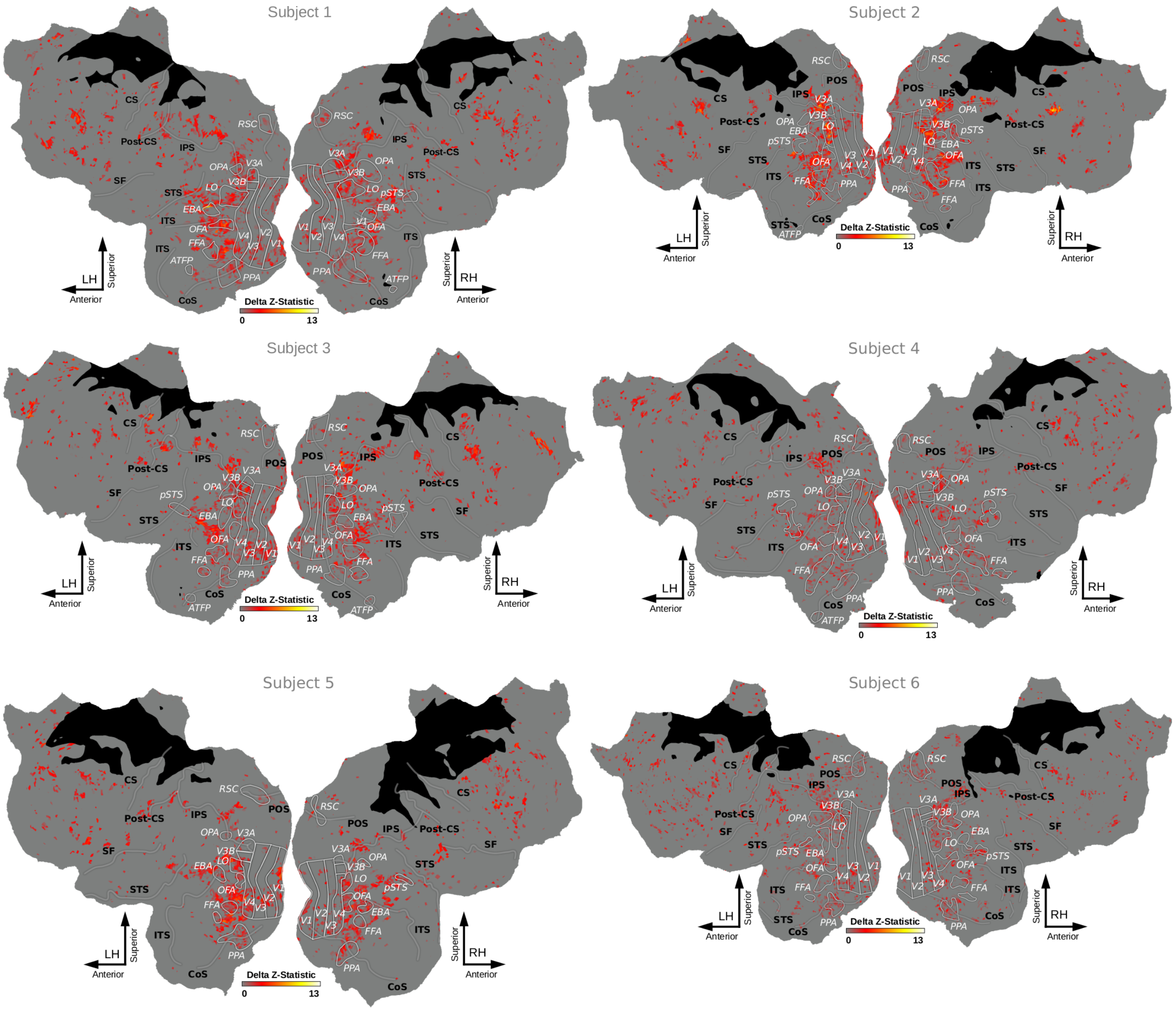

Figure S7. Cortical tuning to stimulus affective features is greater for animate than inanimate stimuli.

This figure shows the relative prediction accuracies for the Semantic with Valence by Arousal for Animate Stimuli (SVAA) model versus the Semantic with Valence by Arousal for Inanimate Stimuli model projected onto cortical maps for each subject. The SVAA model includes features for each semantic category, but only stimuli belonging to animate semantic categories are also labeled for valence and arousal. The SVAI model includes features for each semantic category, but here only stimuli belonging to inanimate semantic categories are labeled for valence and arousal. These subtraction maps effectively reveal the extent to which baseline prediction accuracies achieved by modeling image semantic category are improved to a greater extent by including valence and arousal features for animate stimuli than for inanimate stimuli.

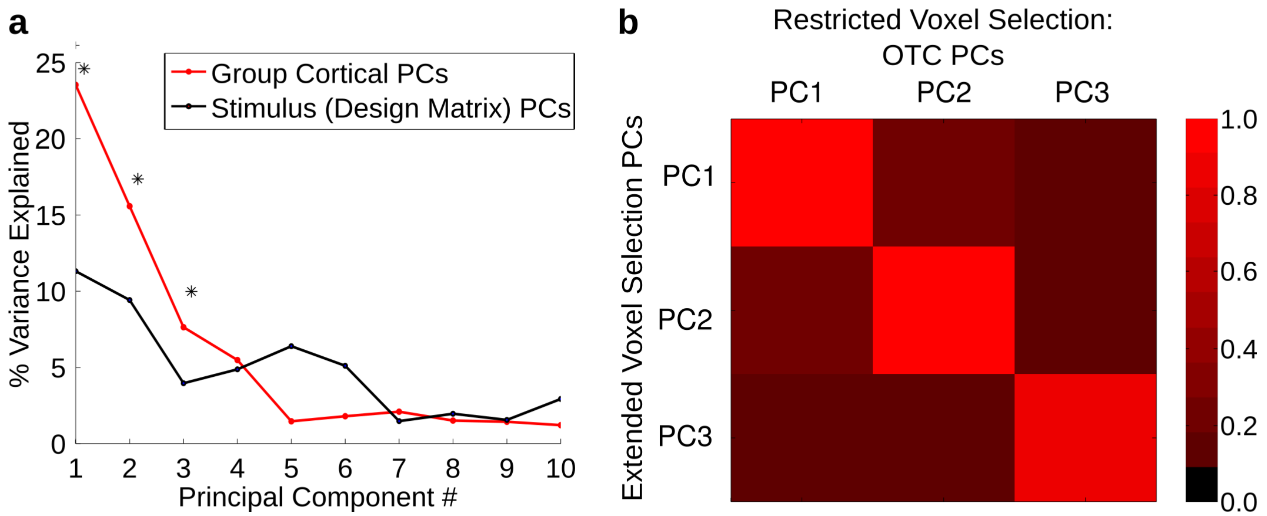

Figure S8. Results of group-level PCA on CSVA model feature weights when voxel selection is expanded to include all cortical voxels where the CSVA model showed a significant fit.

(a) The top three PCs from this analysis explained significantly more variance than the top three stimulus PCs (one-tailed jackknife test, * = p = .03, the smallest possible value given the jackknife test used). This parallels the finding from the PCA reported in the main text where two additional voxel selection criteria were used (voxels within OTC only; CSVA model fit > Semantic Only model fit), see Fig. 4. **(b)** Correlation matrix shows feature loading correlations between the top three Group PCs from this 'expanded' voxel-selection PCA and the 'restricted' voxel selection PCA reported in the main text. Feature loadings for corresponding PCs are correlated as follows: PC1 r(142)=.96, PC2 r(142)=.96, PC3 r(142)=.95.

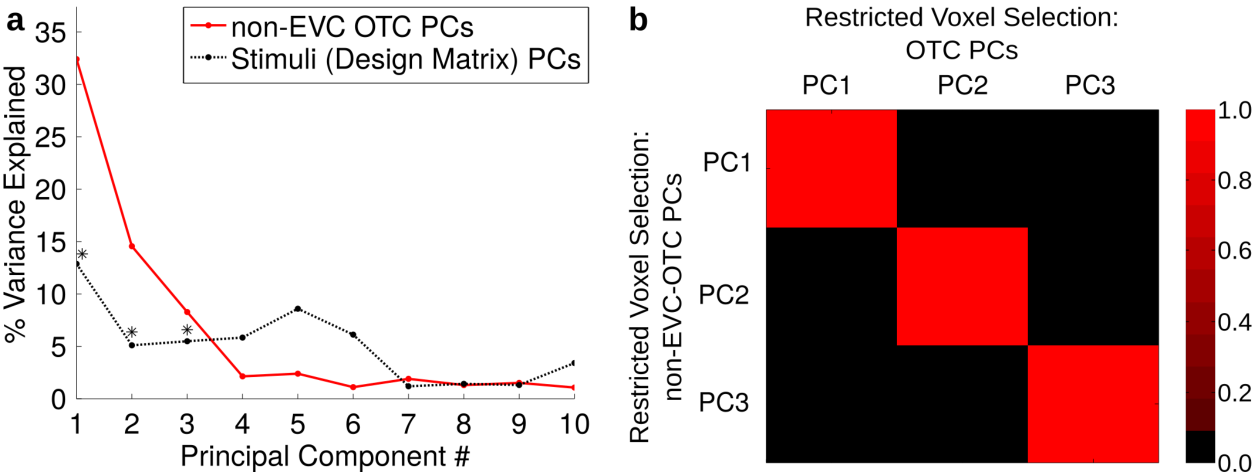

Figure S9. Results of group-level PCA on CSVA model feature weights when voxel selection is further restricted by exclusion of early visual cortex (EVC).
We used retinotopic localizers to specify early visual cortex (EVC) for each participant and to create an OTC ROI with EVC excluded (see Supplementary Methods). We re-conducted PCA on CSVA model weights for voxels within this ROI. As in the CSVA PCA analysis reported in the main text we also excluded voxels where the CSVA model did not outperform the Semantic Only model. (**a**) The top three PCs from this analysis explained significantly more variance than the top three stimulus PCs (one-tailed jackknife test, * = p = .03, the smallest possible value given the jackknife test used). This parallels the finding reported in the main text for OTC with EVC included, see Fig. 4. **(b)** Correlation matrix shows feature loading correlations between the top three Group PCs from this analysis (labeled restricted voxel selection: non-EVC OTC) and the PCA reported in the main text (here labelled restricted voxel selection: OTC). Feature loadings for corresponding PCs are correlated at rs>=0.99.

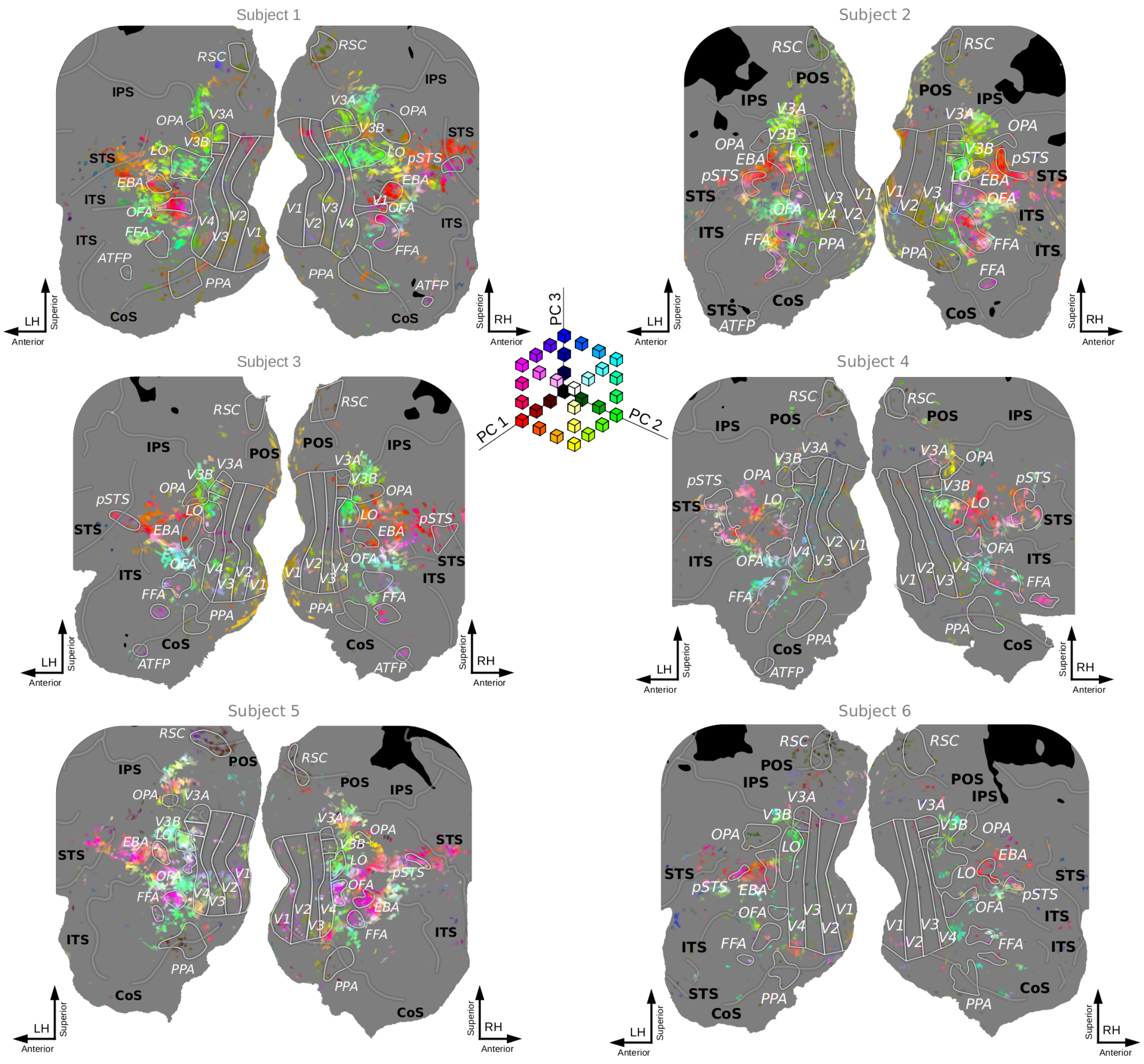

Figure S10. PC scores from the CSVA model projected onto OTC flat maps for each subject.

A principal components analysis (PCA) was conducted on CSVA model feature weights for all OTC voxels where CSVA model fit was significant and superior to that of the Semantic Only model. PC scores were calculated as the product of CSVA feature weights for a given voxel by feature loadings for each PC. Here, a RGB color space is used to map PC scores onto cortex (red = scores on PC1, green = scores on PC2, blue = scores on PC3). PC scores are thresholded at 6 standard deviations above and below 0 with values beyond the threshold given the maximal (or minimal) color channel value. Consistent spatial structure of voxel-wise tuning to the top three group PCs is observed across subjects. Note. Areas where MRI data was not acquired are shown in black. Both voxels where the CSVA model did not fit significantly and those where the CSVA model fit significantly but did not outperform the semantic only model were excluded from the PCA (these voxels are shown in grey). PCA maps using CSVA model feature weights from all voxels where the CSVA model fit significantly are given in Fig. S11.

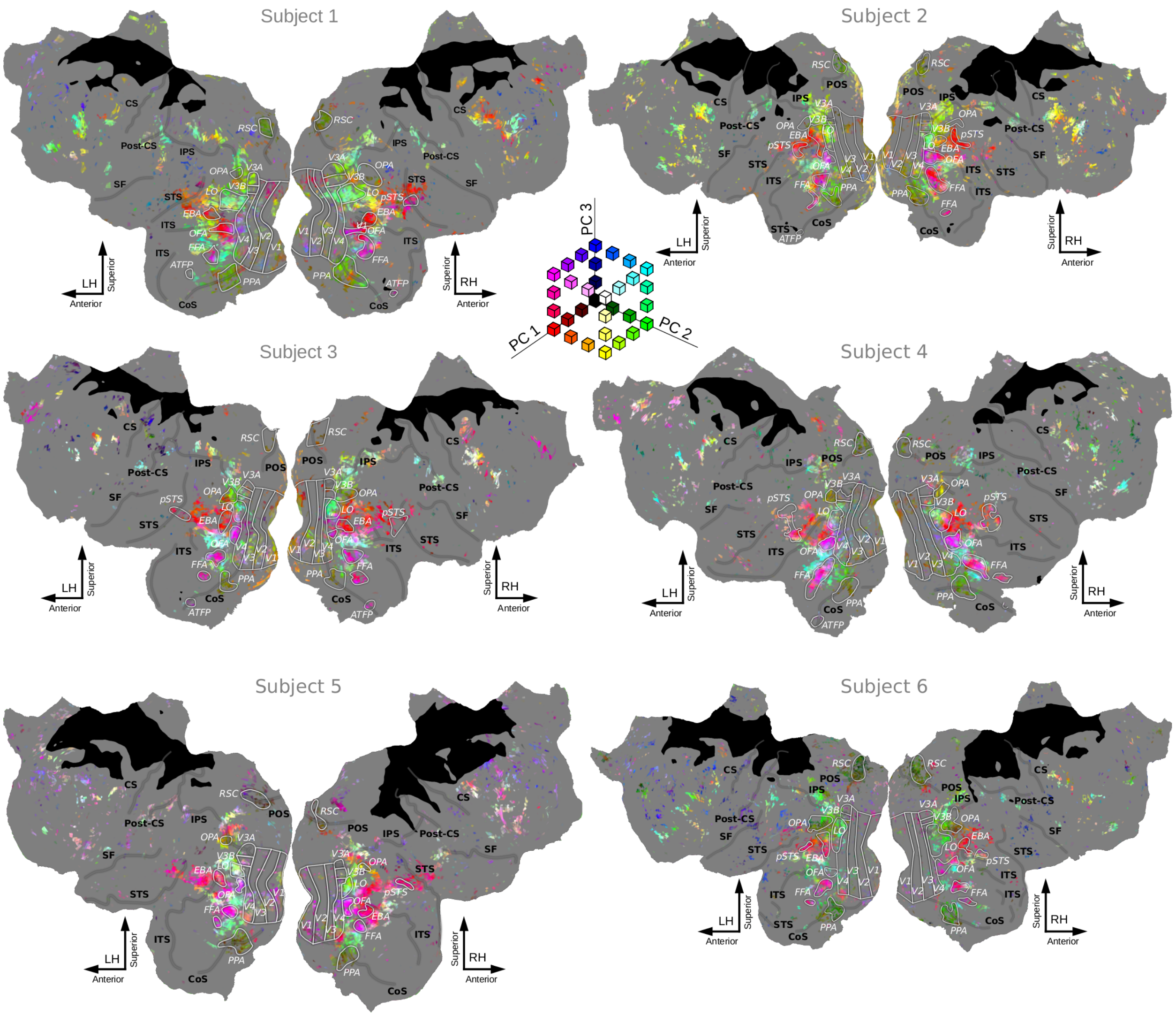

Figure S11. PC scores from PCA on CSVA model weights, across all cortical voxels where the CSVA model fits, projected onto cortical flat maps for each subject.

Here, we map PC scores onto cortex using the top three PCs from PCA on CSVA model weights across all cortical voxels where the CSVA model fit was significant. As illustrated in fig. S8, when voxel selection is expanded in this manner, the top three PCs are highly correlated with those from the OTC analysis reported in the main text (rs>.95). As in fig. 6a and fig. S10, a RGB color space is used to map PC scores onto cortex (red = scores on PC1, green = scores on PC2, blue = scores on PC3). PC scores are thresholded at 6 standard deviations above and below 0.

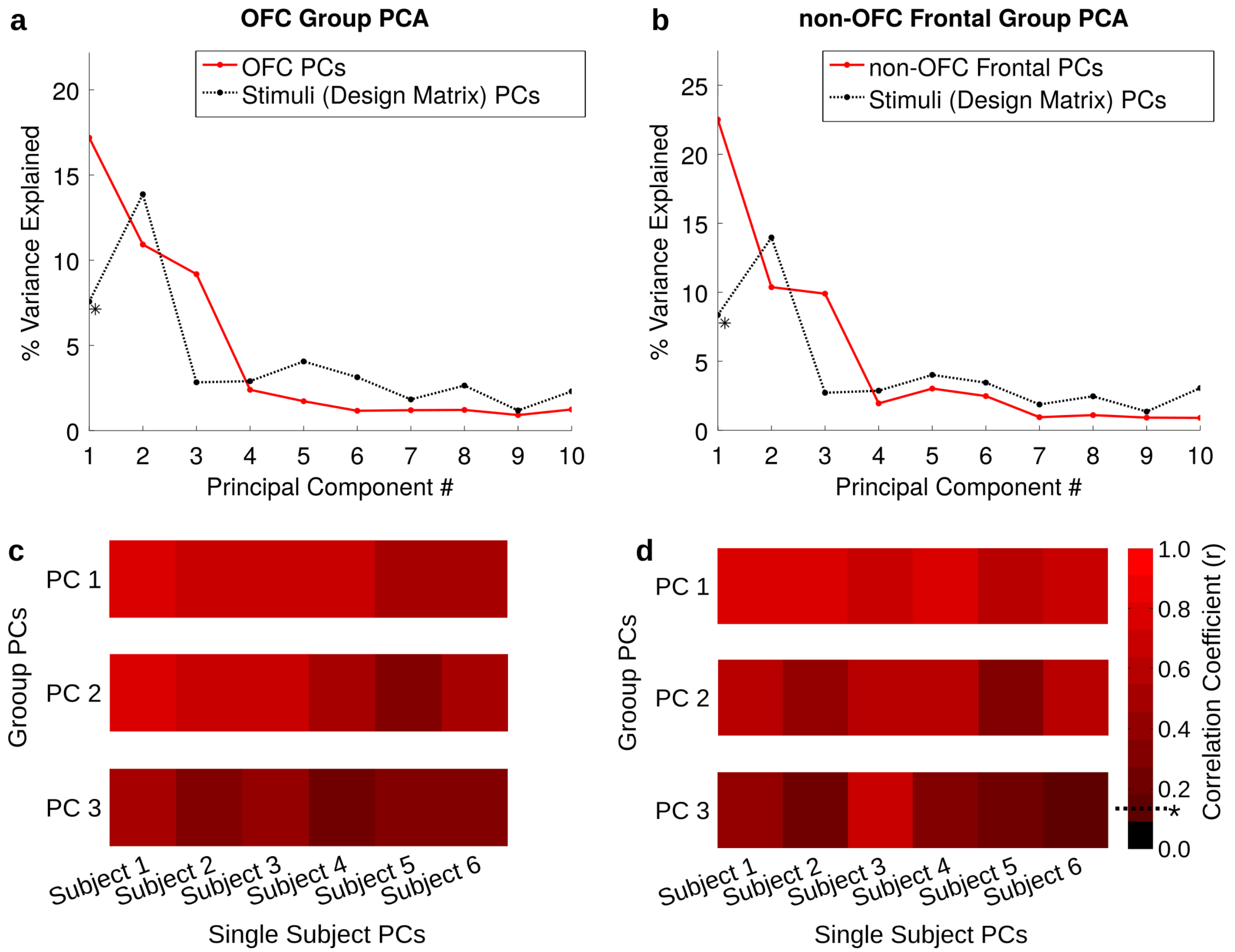

Figure S12. PCA results for Orbital frontal cortex (OFC) and non-OFC frontal cortical ROIs. The orbital frontal cortex (OFC) has been especially implicated in the representation of stimulus affective value. Hence, we divided frontal cortex into two regions of interest: OFC and non-OFC frontal cortex (see Supplementary Methods for frontal ROI definition). We then conducted PCA on CSVA model weights across all voxels within each of these ROIs where the CSVA model fit significantly. **(a,b)** In both OFC **(a)** and non-OFC frontal cortex **(b)**, only the top group PC explained significantly more variance than the corresponding PC from PCA conducted directly on the stimulus features themselves (one-tailed jackknife test, * = p =0.03, the smallest possible value given the jackknife test used) **(c,d)** Results of a leave-one-out cross validation analysis of the similarity in feature loadings between individual subject PCs and group PCs. The correlation matrices presented give the correlation of feature loadings for the top three PCs extracted from PCA conducted on each individual subject's data and the top 3 PCs from the group-level PCA conducted on the data from all remaining subjects. In both OFC **(c)** and the non-OFC frontal ROI **(d)**, the top two group PCs show similarity to individual level PCs across participants. This is less true for the third PC, here correlations between the group and single subject PC loadings are not significant for several subjects. Note. R values above the dotted line on the heat bar to the far right are significant at p=0.05. Significance was calculated by permutation test (see Methods).

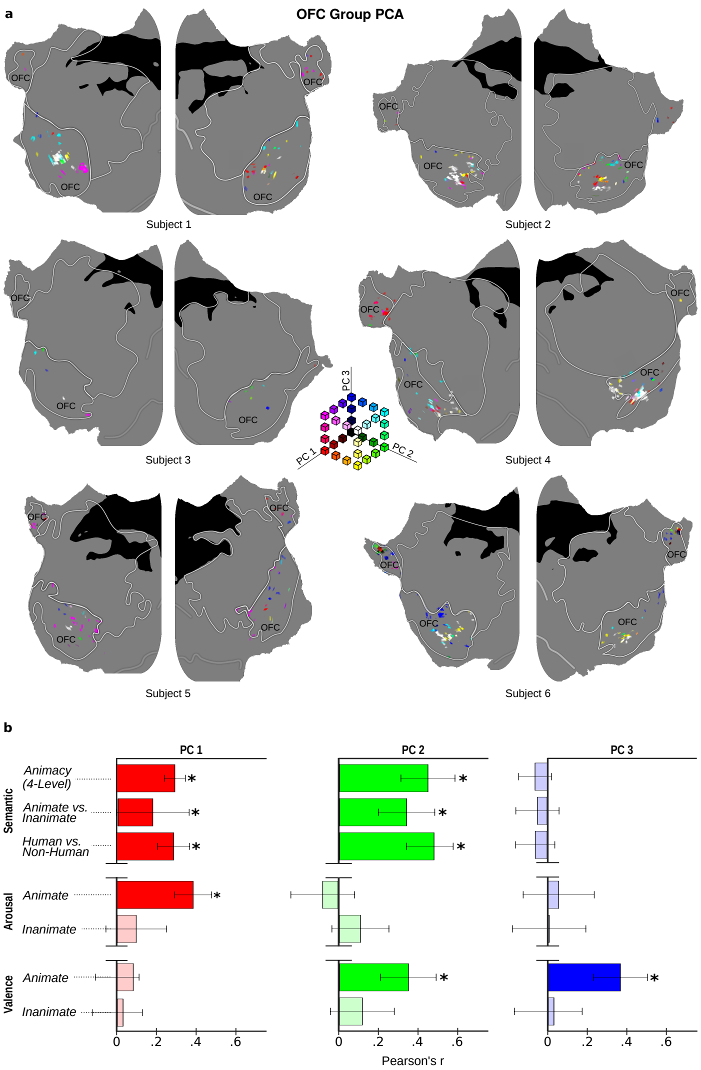

Figure S13. Top 3 OFC group PCs: flat-map projections and hypothetical dimension correlations. (a) Scores for the top three PCs from the group-level PCA conducted across voxels within OFC are projected onto frontal flat-maps for each subject. The OFC ROI is outlined in white and labeled. As in Fig. S10, a RGB color space is used to map PC scores onto cortex (red = scores on PC1, green = scores on PC2, blue = scores on PC3), and PC scores are thresholded at 6 standard deviations above and below 0. Only voxels where the CSVA model fits significantly are shown, these are relatively scarce within OFC. In contrast to OTC, these OFC PC score maps reveal little consistent spatial structure in tuning to affective or semantic image information as captured by the CSVA model. A small number of voxels (<3 per subject) falling outside of the OFC ROI due to interpolation error were greyed out for visualization purposes. **(b)** To aid in PC interpretation, we present Pearson's r correlations for feature loadings (n=144) on the top three PCs with feature loadings on the theoretical dimensions of interest described in the main text (see Figure 5). Bootstraping was used with 5000 resamples to perform one-tailed significance tests of correlation coefficients. Bars show pearson correlation coefficients (r) +/- sd calculated from bootstrap samples for each of the top three PCs (left to right) against each theoretical dimension (y axis). Saturated color and * indicates correlations signifcant at $p < 0.05$, transparent colors indicate correlations that are not significant.

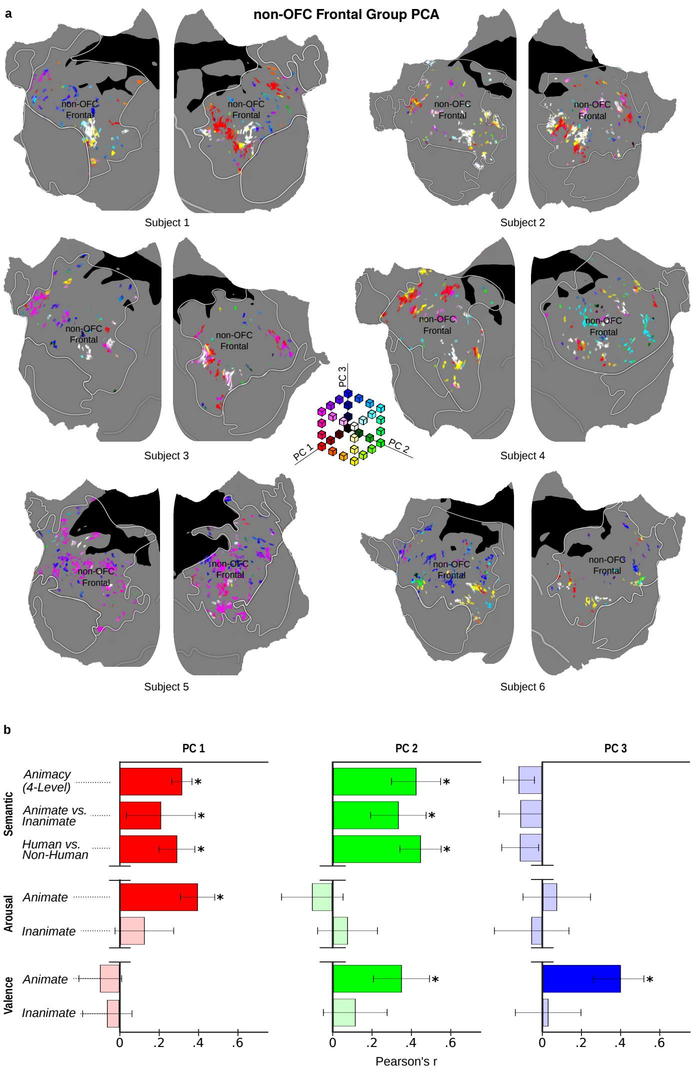

Figure S14. Top 3 non-OFC frontal group PCs: flat-map projections and hypothetical dimension correlations. (a) Scores for the top three PCs from the group-level PCA conducted across voxels within the non-OFC frontal ROI are projected onto frontal flat-maps for each subject. The non-OFC frontal ROI is outlined in white and labeled. As in Fig. S10, a RGB color space is used to map PC scores onto cortex (red = scores on PC1, green = scores on PC2, blue = scores on PC3), and PC scores are thresholded at 6 standard deviations above and below 0. Only voxels where the CSVA model fit significantly are shown. A small number of voxels (<3 per subject) falling outside of the non-OFC Frontal ROI due to interpolation error were greyed out for visualization purposes. Within the non-OFC frontal ROI, tuning to affective or semantic image information as captured by the CSVA model shows little consistency in spatial organization across subjects. **(b)** To aid in PC interpretation, we present Pearson's r correlations for feature loadings (n=144) on the top three PCs with feature loadings on the theoretical dimensions of interest described in the main text (see Figure 5). Bootstraping was used with 5000 resamples to perform one-tailed significance tests of correlation coefficients. Bars show pearson correlation coefficients (r) +/- sd calculated from bootstrap samples for each of the top three PCs (left to right) against each theoretical dimension (y axis). Saturated color and * indicates correlations signifcant at $p < 0.05$, transparent colors indicate correlations that are not significant.

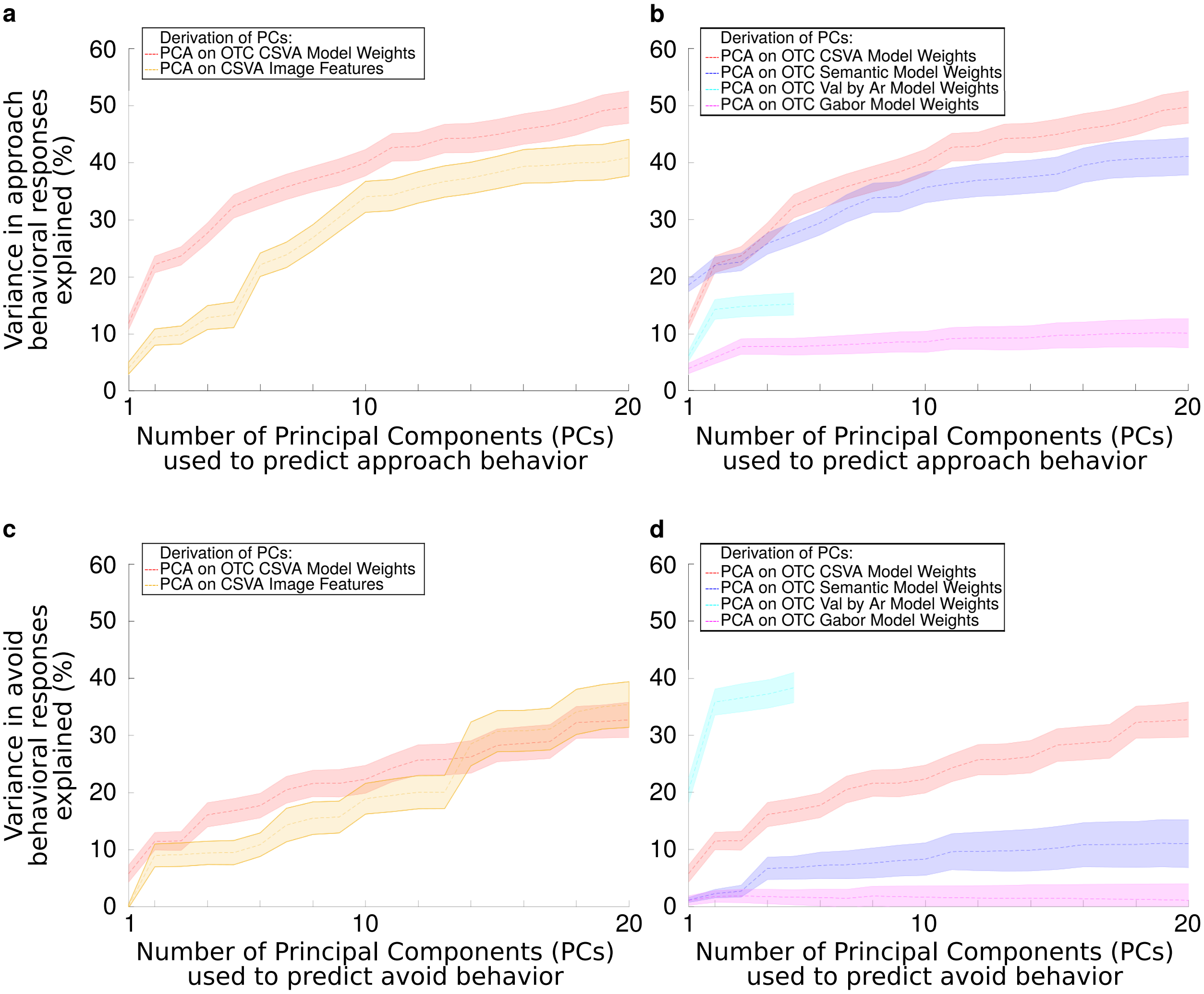

Figure S15. OTC tuning ability to predict the approach behavior(s) (panels a and b) and avoidance behavior(s) (panels c and d) that best matches a given image. This figure complements Figure 9. **Left:** The extent to which OTC tuning to emotional images, as captured by CSVA model group-level PC scores (red line) predicts behavioral responses selected, across images (n=1440), was examined separately for approach behaviors (**a**) and avoidance behaviors (**c**). The percentage of out-of-sample variance in behavioral responses explained (y axis) is plotted against the number of PCs included as predictors in an ordinary least squares regression analysis. The out-of-sample variance in behavioral responses explained using PCs derived directly from PCA on CSVA image features, across images is given by the yellow line. Across all levels of dimensionality considered (nu. of PCs=1 to 21), OTC tuning to CSVA features predicted approach responses significantly better than components from PCA conducted directly on the features themselves. This superiority was less evident but present at low numbers of PCs (<10) for avoidance behaviors. **Right:** Using the analyses including only approach behaviors (**b**) or only avoidance behaviors (**d**), these two panels present a comparison of the out-of-sample variance in behavioral responses explained by PCs obtained from PCA on OTC voxel-wise feature weights for the CSVA model (red) versus (i) the Gabor model (pink) (ii) the Semantic Only model (dark blue) and (iii) the Valence by Arousal model (light blue). Note that the

Valence by Arousal model only includes six features, so that is also the maximum number of PCs that can be extracted for this model. The Gabor model performed poorly in the prediction of both approach and avoidance behavioral responses. In differentiating within approach behaviors **(b)**, the CSVA model showed generally superior performance, followed by the Semantic Only model. Interestingly, the Valence by Arousal model performed best in predicting within avoidance behaviors **(d)**. Note. Variance in behavioral responses explained (y axes) is calculated using leave one out cross validation and scaled by explainable variance (see methods). Error bands around each dotted line represent 95% confidence intervals.

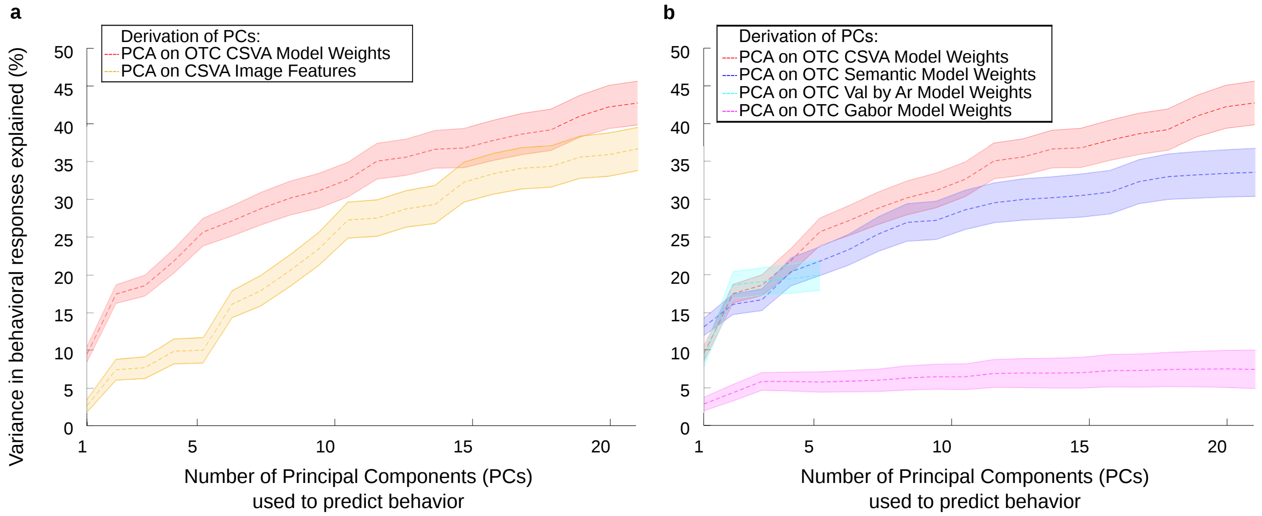

Figure S16. OTC tuning prediction of behavioral responses to emotional natural stimuli: response categories that do not entail overt behavior removed. Here, we repeated the analysis reported in the main manuscript with the exception that we removed the three categories that did not entail an overt behavioral response, namely: be supported by, empathy for joy, empathy for suffering. The results parallel those shown in Figure 9. Both panels show percentage of out-of-sample variance in behavioral responses explained across images (n=1440) plotted (y axis) against the number of PCs included as predictors in an ordinary least squares regression analysis (n=1 to 21, x axis). The error bands around each dotted line represents the 95% confidence interval. (a) OTC tuning to CSVA features (as captured by CSVA model group-level PC scores) predicted out of sample variance in behavioral responses significantly better than components from PCA conducted directly on the features themselves. (b) Both PCs from PCA on the Semantic Only (dark blue) and Valence by Arousal (light blue) model feature weights, across OTC voxels, outperform PCs from PCA on Gabor (pink) model weights, across OTC voxels, in predicting behavior. However, their maximal prediction of behavior (at n=21 and n=6 PCs respectively) is significantly less than that achieved by PCs from PCA on CSVA model feature weights across OTC using an equivalent number of components. Note. Variance in behavioral responses explained (y axes) is calculated using leave one out cross validation and scaled by explainable variance (see methods).

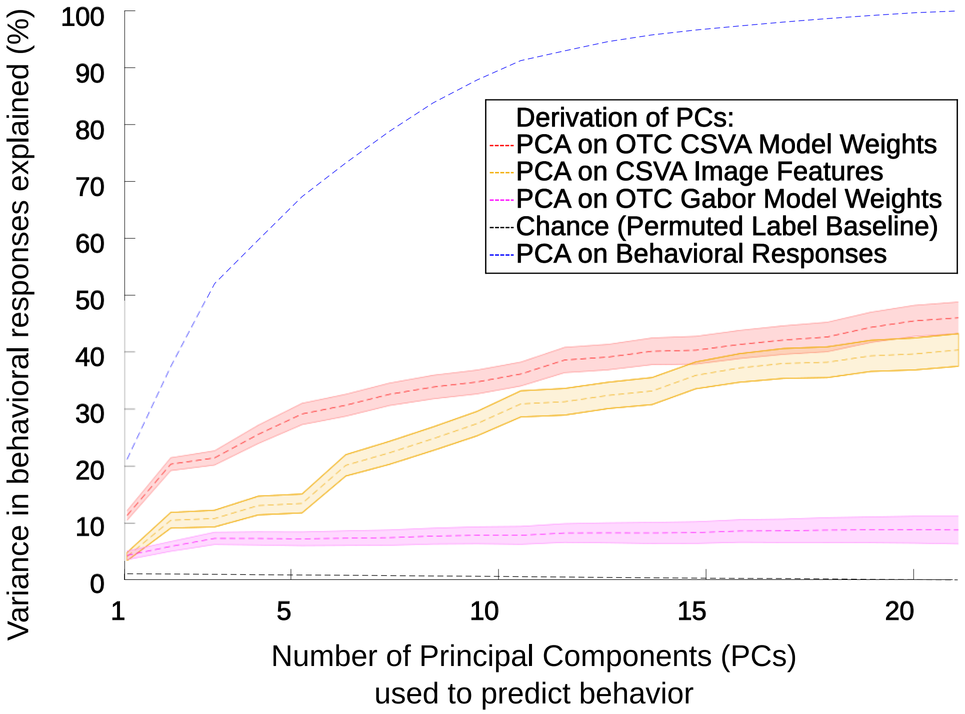

Figure S17. Prediction of behavioral responses to emotional natural stimuli: upper bounds and chance performance.

This figure aims to contextualize the extent to which OTC tuning to emotional natural images, as captured by CSVA group-level PC scores, is able to predict the behavioral responses selected for each image by a novel group of participants, across images (n=1440).The percentage of out-of-sample variance in behavioral responses explained (y axis) is plotted against the number of PCs included as predictors in an ordinary least squares regression analysis (dotted red line). Dotted lines of other color represent the performance of PCs derived in alternate fashions. As in Fig 9a, the performance of PCs derived directly from PCA on CSVA image features is shown by the yellow line and that of PCs obtained from PCA on OTC feature weights for a Gabor model is given by the pink line. In each case, the error band around the dotted line represents the 95% confidence interval. Two new lines are included. The black dotted line represents chance performance obtained by permutation of the behavioral responses across images. Specifically, the behavioral responses linked to each image were shuffled across images (i.e. the rows of the behavioral response matrix shown in Fig 8 were shuffled, with the images held constant). This was repeated 100 times. OTC CSVA PC scores for each image were calculated as normal (using the inner product of each image's feature vector with each of the PC loading vectors from the group-level PCA of CSVA model feature weights, across OTC voxels). These PC scores were used to predict the permuted behavioral responses. As in the main analysis, we used leave-one(image)-out cross-validation (LOOCV) and scaled LOOCV R^2 values by the total explainable variance in behavioral responses. The blue dotted line gives an upper bound to prediction performance with increasing PCs. Here, the PCs used are from PCA on the behavioral ratings themselves. It can be seen that the top component explains about 20% of variance in responses, the top 3 about 50% and over 15 components are needed to explain close to 100% of the variance in behavioral responses across images.

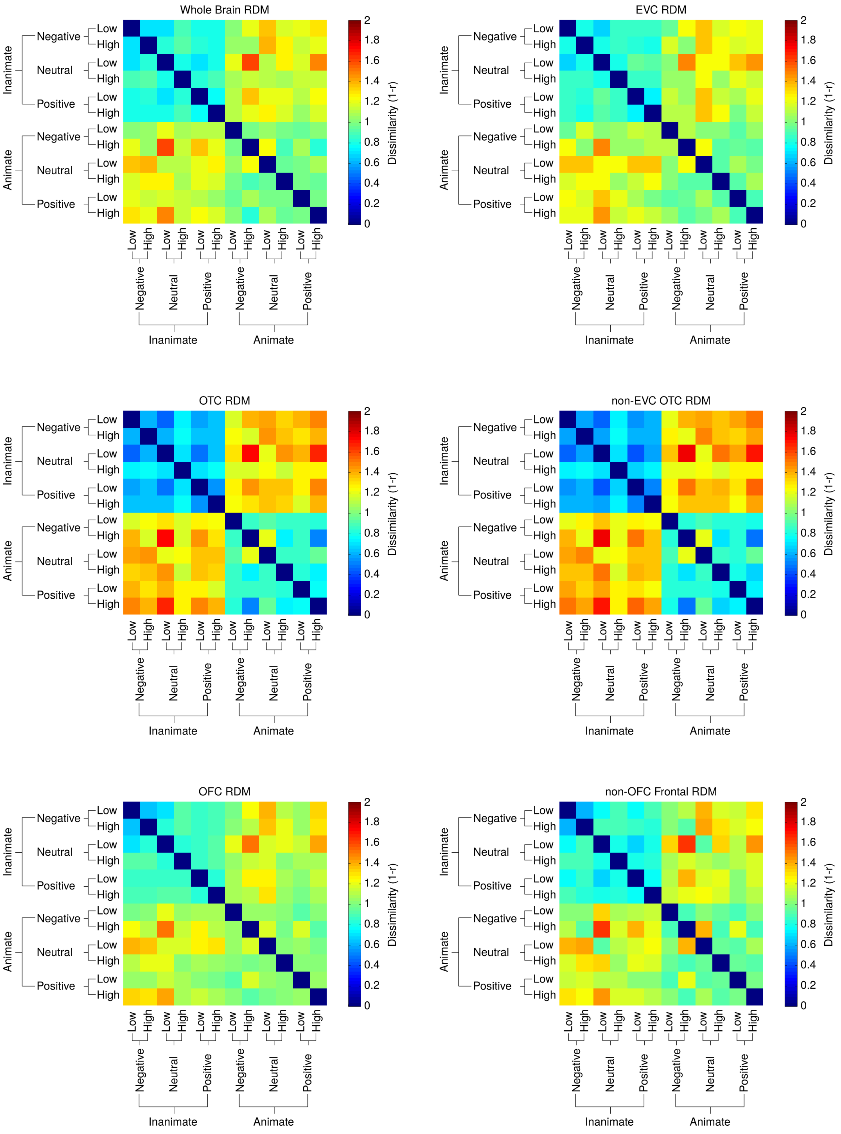

Figure S18. Dissimilarity of Stimulus Representation within ROIs as revealed by Group-Averaged Brain RDMs. The matrices shown here give group-averaged dissimilarity of stimulus representation by stimulus condition for the following ROIs: whole brain, early visual cortex (EVC), OTC, Non-EVC OTC, OFC, non-OFC Frontal. Higher dissimilarity values are represented by hotter (yellow-red) colors and lower dissimilarity values are represented by colder (blue-green) colors. A higher dissimilarity value is the result of lower Pearson's correlation between the multivoxel pattern of activity of two stimulus conditions, across the given region, and vice versa for lower dissimilarity values. As has been reported previously (Kriegeskorte et al., 2008), across all ROIs, and the whole brain, an animate vs. inanimate divide can be seen, although it is much more prominent within the non-EVC OTC, as would be expected from previous findings. Stimuli are more differentiated by affective properties within the non-EVC OTC than in other regions but some differentiation by affective properties is still discernable within frontal regions (e.g. neutral low arousal inanimate stimuli show the highest dissimilarity to positive and negative high arousal animate stimuli)

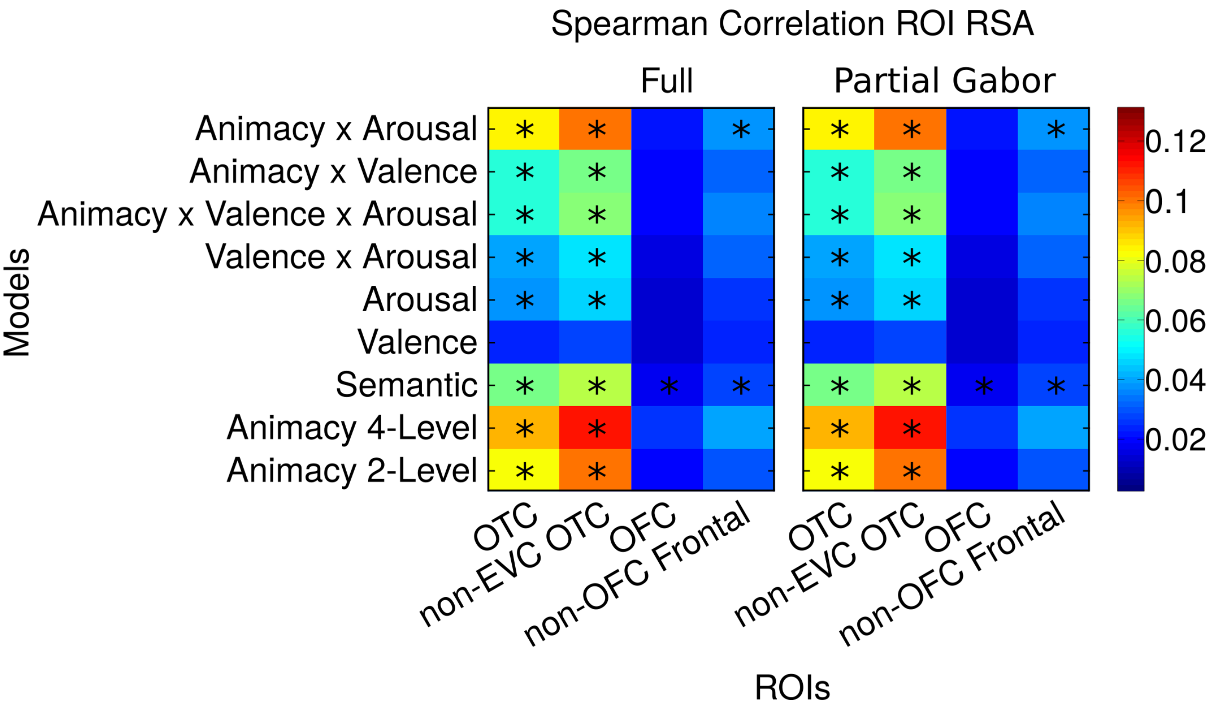

Figure S19. Correlation matrixes showing full (left) and partial (right) Spearman rank correlations between brain RDMs (columns) and model RDMs (rows). See Table S5 for model RDM details. The matrix on the left gives full Spearman rank correlations, across stimuli (n=1440). The matrix on the right gives partial Spearman rank correlations, controlling for correlations between both the brain and model RDM in question with a Gabor model RDM. The color of each matrix cell gives the average strength of brain RDM – model RDM correlation across subjects (see heat map to far right). A one-tailed t-test of Fisher transformed correlations, across subjects, was used to determine the significance of each brain RDM – model RDM correlation (an asterisk signifies p<.05, bonferroni corrected).

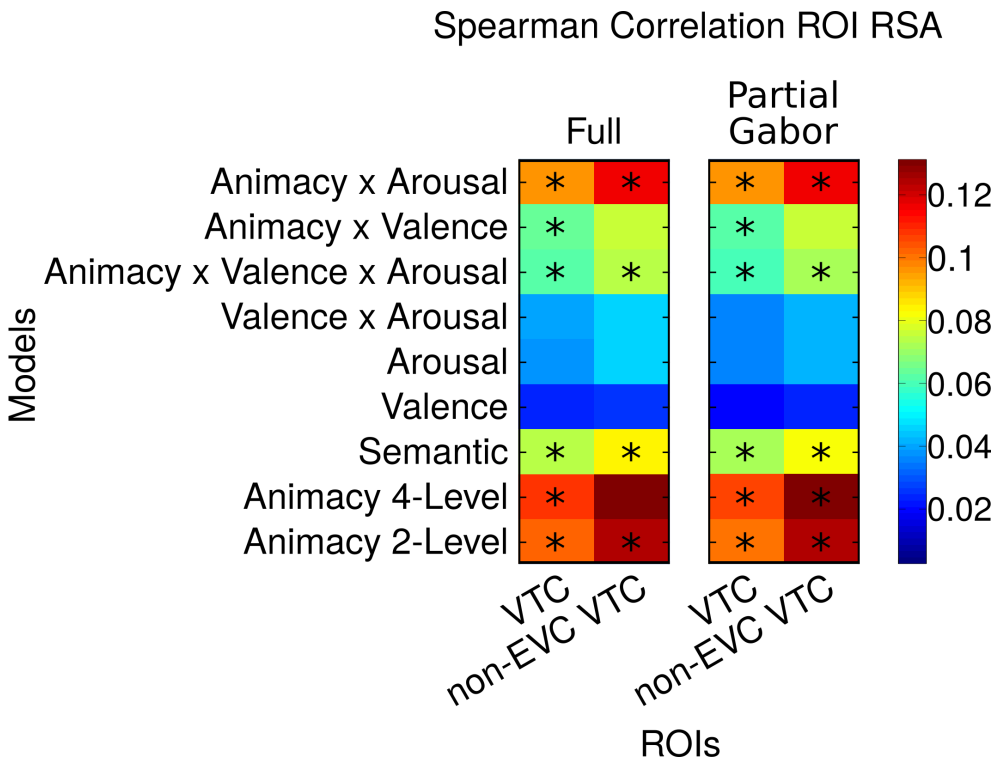

Figure S20. Correlation matrixes showing full (left) and partial (right) Spearman rank correlations between ventro-temporal cortical RDMs (columns) and model RDMs (rows). To allow more direct comparability with prior work[S6], in the analyses reported here we replaced our OTC and non-EVC OTC ROIs with a ventro-temporal cortical (VTC) ROI defined following [S6] as well as a corresponding ROI excluding early visual cortex (non-EVC VTC). As in Figure S19, the matrix on the left gives full Spearman rank correlations (across estimation images). The matrix on the right gives partial Spearman rank correlations, controlling for correlations between both the brain and model RDM in question with a Gabor model RDM. The color of each matrix cell gives the average strength of brain RDM – model RDM correlation across subjects (see heat map to far right). A one-tailed t-test of Fisher transformed correlations, across subjects, was used to determine the significance of each brain RDM – model RDM correlation (an asterisk signifies $p<.05$, bonferroni corrected).
