# Supplementary material for: Occipital-temporal cortical tuning to semantic and affective features of natural images predicts associated behavioral responses
Source: Nat Commun. 2024 Jul 9;15:5531. doi: 10.1038/s41467-024-49073-8 (PMC11233618; doi:10.1038/s41467-024-49073-8)
Supplement: Supplementary file 2 — Reporting Summary [file 41467_2024_49073_MOESM2_ESM.pdf]

nature portfolio

Corresponding author(s): Sonia Bishop
Last updated by author(s): Apr 28, 2024

Reporting Summary

Nature Portfolio wishes to improve the reproducibility of the work that we publish. This form provides structure for consistency and transparency in reporting. For further information on Nature Portfolio policies, see our Editorial Policies and the Editorial Policy Checklist.

Statistics

For all statistical analyses, confirm that the following items are present in the figure legend, table legend, main text, or Methods section.

n/a	Confirmed	
☐	☒	The exact sample size (n) for each experimental group/condition, given as a discrete number and unit of measurement
☐	☒	A statement on whether measurements were taken from distinct samples or whether the same sample was measured repeatedly
☐	☒	The statistical test(s) used AND whether they are one- or two-sided<br>*Only common tests should be described solely by name; describe more complex techniques in the Methods section.*
☐	☒	A description of all covariates tested
☐	☒	A description of any assumptions or corrections, such as tests of normality and adjustment for multiple comparisons
☐	☒	A full description of the statistical parameters including central tendency (e.g. means) or other basic estimates (e.g. regression coefficient) AND variation (e.g. standard deviation) or associated estimates of uncertainty (e.g. confidence intervals)
☐	☒	For null hypothesis testing, the test statistic (e.g. F, t, r) with confidence intervals, effect sizes, degrees of freedom and P value noted<br>*Give P values as exact values whenever suitable.*
☒	☐	For Bayesian analysis, information on the choice of priors and Markov chain Monte Carlo settings
☒	☐	For hierarchical and complex designs, identification of the appropriate level for tests and full reporting of outcomes
☐	☒	Estimates of effect sizes (e.g. Cohen's d, Pearson's r), indicating how they were calculated

Our web collection on statistics for biologists contains articles on many of the points above.

Software and code

Policy information about availability of computer code

Data collection	Task presentation was conducted using the Python Vision Egg toolbox, version 1.2.1. Physiological data (pulse oximetry, respiration) was collected using using Biopac AcqKnowledge proprietary software v4.2. MRI data was collected on a Siemens MAGNETOM Trio 3T scanner using Siemen's B19 proprietary software. Image behavioral response ratings were collected using the Amazon Mechanical Turk web interface.
Data analysis	Following conversion from DICOM to NIFTI format, the fMRI data were intra- and inter-run aligned using the Statistical Parameter Mapping toolbox (SPM8). Non-brain tissue was excluded from further analysis using the FSL Brain Extraction Tool (BET). The cortical surface of each subject was reconstructed from anatomical data using Freesurfer. The Gallant lab voxelwise modeling framework was used for regression and PCA analyses (see https://gallantlab.org/voxelwise_tutorials; for modeling choices and parameter specifications also see the readme.txt file in https://osf.io/b5pxu/). Flatmap visualizations were created in Python using the PyCortex package (https://github.com/gallantlab/pycortex). MVPA Representational Similarity Analyses (as reported in the supplements) were conducted using the CosMoMVPA toolbox (https://www.cosmomvpa.org/). Extraction of physiological regressors was conducted using the Physiological Log Extraction for Modeling (PhLEM) Matlab Toolbox (https://github.com/timothyv/Physiological-Log-Extraction-for-Modeling--PhLEM--Toolbox).

For manuscripts utilizing custom algorithms or software that are central to the research but not yet described in published literature, software must be made available to editors and reviewers. We strongly encourage code deposition in a community repository (e.g. GitHub). See the Nature Portfolio guidelines for submitting code & software for further information.

Data

Policy information about availability of data

All manuscripts must include a data availability statement. This statement should provide the following information, where applicable:

- Accession codes, unique identifiers, or web links for publicly available datasets
- A description of any restrictions on data availability
- For clinical datasets or third party data, please ensure that the statement adheres to our policy

The fmri data for all subjects is available together with the CSVA model feature matrices via the Open Science Framework database - https://osf.io/b5pxu/. For data privacy reasons, structural images have been skull stripped prior to sharing.

Research involving human participants, their data, or biological material

Policy information about studies with human participants or human data. See also policy information about sex, gender (identity/presentation), and sexual orientation and race, ethnicity and racism.

Reporting on sex and gender	Participants' gender: 4 were female, 2 were male. This was self-reported. We did not ask participants for sex at birth. To the best of our knowledge gender matched sex at birth. We do not report data separately by gender as with our small sample size this might risk identifying participants.
Reporting on race, ethnicity, or other socially relevant groupings	We did not request participants to report their race or ethnicity.
Population characteristics	Participants were aged between 21 and 26 years, with a mean of 24 years.
Recruitment	Participants were recruited through flyers and direct email advertisements at the University of California, Berkeley. No participants were members of the PI's lab.
Ethics oversight	The study was approved by the Institutional Review Board of the University of California, Berkeley. All participants provided written informed consent.

Note that full information on the approval of the study protocol must also be provided in the manuscript.

Field-specific reporting

Please select the one below that is the best fit for your research. If you are not sure, read the appropriate sections before making your selection.

☒ Life sciences ☐ Behavioural & social sciences ☐ Ecological, evolutionary & environmental sciences

For a reference copy of the document with all sections, see nature.com/documents/nr-reporting-summary-flat.pdf

Life sciences study design

All studies must disclose on these points even when the disclosure is negative.

Sample size	Sample size (n=6 subjects) is based on prior voxel-wise modeling experiments with similar designs and stimuli (see Lescroart et al., 2015; Huth et al., 2016.) As in primate electrophysiology or human psychophysics, power is maximized within subjects as opposed to between subjects. Here, we collected 50 imaging runs per participant across 6 fmri sessions; giving 345 minutes of data acquisition per subject and responses to over 1600 natural images per subject. By using leave one out cross validation we are able to determine the correlation between the principle dimensions of OTC tuning to image features for each subject and those for the remainder of the group. This takes advantage of our considerable statistical power within subjects while also allowing us to establish replicability of dimensional spaces across participants.
Data exclusions	Exclusion criteria for participation included current receipt of psychoactive medication, neurological illness, or contraindications for MRI participation. All subjects had normal or corrected-to-normal vision. At the time of data collection, one of the MRI scanner gradient coils' bolts came loose causing arcing and spiking in the data. As a result we had to exclude data from 4 participants. A fifth participant was excluded due to sleeping during the study.
Replication	Statistical models were estimated and validated within each individual subject using separate estimation (training) and validation (test) data-sets. By using leave one out cross validation we are able to determine the correlation between the principle dimensions of OTC tuning to image features for each subject and those for the remainder of the group (i.e. we can see if representational spaces replicate across subjects). For our analysis of appropriate behavioral responses we used leave one out cross-validation (LOOCV).
Randomization	No group allocation was conducted.
Blinding	N/A (Blinding is only relevant when there are different experimental conditions either across or within subjects - here every stimulus has multiple features which are modeled simultaneously so there is not anything which one would 'blind')

Reporting for specific materials, systems and methods

We require information from authors about some types of materials, experimental systems and methods used in many studies. Here, indicate whether each material, system or method listed is relevant to your study. If you are not sure if a list item applies to your research, read the appropriate section before selecting a response.

Materials & experimental systems

n/a	Involved in the study
☒	☐ Antibodies
☒	☐ Eukaryotic cell lines
☒	☐ Palaeontology and archaeology
☒	☐ Animals and other organisms
☒	☐ Clinical data
☒	☐ Dual use research of concern
☒	☐ Plants

Methods

n/a	Involved in the study
☒	☐ ChIP-seq
☒	☐ Flow cytometry
☐	☒ MRI-based neuroimaging

Magnetic resonance imaging

Experimental design

Design type

Event-related task-based design.

Design specifications

Subjects completed six fMRI sessions. Four 9.5 minute retinotopy scans (two with clockwise/counterclockwise wedges and two with expanding/contracting rings; Huth et al., 2012) were completed within session 1. Each of the subsequent five sessions comprised performance of the main task. In each of these sessions, participants completed six model estimation scans of 7.5 minute duration and four model validation scans of 6 minute duration. A structural scan was also acquired at the beginning of each sessions. Trials were 4 seconds each: 1 second of stimulus presentation and 3 seconds of rest.

Behavioral performance measures

While viewing the images, subjects performed one of two tasks. Four subjects (1,3, 5 and 6) were asked to categorize the valence of each image as negative, neutral, or positive. To control for effects of task, two subjects (2, 4) performed an alternative semantic categorization task, categorizing each image as human, animal, object, food or building/scene. No performance metric was formally used within our analyses.

Acquisition

Imaging type(s)

Functional T2*-weighted images and anatomical T1-weighted images were acquired.

Field strength

3T

Sequence & imaging parameters

Functional: repetition time = 2 s, echo time = 34 ms, flip angle = 74 degrees, voxel size = 2.6 x 2.6 x 3 mm^3, field of view = 224 x 224 mm^2.
Anatomical: 1 x 1 x 1 mm^3 voxel size and 256 x 212 x 256 mm^3 field of view using a three-dimensional T1-weighted MP-RAGE sequence.

Area of acquisition

We prescribed 25 axial slices to cover all of the temporal and occipital cortices, and as much of frontal and parietal cortices as possible.

Diffusion MRI ☐ Used ☒ Not used

Preprocessing

Preprocessing software

Following conversion from DICOM to NIFTI format, the fMRI data were intra- and inter-run aligned using the Statistical Parameter Mapping toolbox {SPM8). Non-brain tissue was excluded from further analysis using the FSL Brain Extraction Tool (BET). The cortical surface of each subject was reconstructed from anatomical data using Freesurfer. Further analyses were performed using MATLAB (r2012b)

Normalization

The BOLD timeseries were normalized to have zero mean and unit variance. Data were neither transformed to common space (spatially normalized) nor spatially smoothed in order to retain maximal resolution for our voxel-wise modelling.

Normalization template

Individual subjects' data were not normalized to a template. Only within-subject analyses were conducted at the voxel level.

Noise and artifact removal

Low-frequency drifts in voxel responses were estimated using a 120s long cubic Savitzky-Golay filter and removed from the BOLD timeseries for each run. Motion parameters estimated during preprocessing (6 parameters for 3D displacement and rotation) were regressed out of the BOLD responses prior to model estimation or validation. We

repeated all analyses with physiological noise {pulse and respiration) regressed out of the BOLD responses, yielding very similar results (Supplementary Fig. 3). Pulse oximetry and respiration data were collected using a Biopac recording system (Biopac MP150 Data Acquisition Unit, Biopac UIM100C with Nonin 8600FO for pulse oximetry, and Biopac RSP100C with Biopac TSD221 for respiration). Regressors were constructed to capture phase and variation in pulse and respiration signals, as estimated using the PhLEM Toolbox.

Volume censoring

The first 5 volumes of each run were discarded. Diagnostics were run on the BOLD time series from each scan. Following an approach similar to that adopted by Power and colleagues (Power at al., 2012), and Carp {Carp, 2013), bad volumes (with unusually high changes in mean whole-brain signal intensity) were identified using the SPM time-series diagnostic tool tsdiffana.m (http://imaging.mrc-cbu.cam.ac.uk/imaging/DataDiagnostics). Among other indices, this tool calculates the mean square difference of voxel-wise signal intensities between each volume (n) and the previous volume (n-1) and divides this by the mean signal across the whole volume averaged over the whole time-series. Volumes (both n and n-1) were rejected using an absolute cutoff {the recommended default of 10) as this handles differences between subjects in the noisiness of data better than a within-subject percentile cut off. Bad volumes tended to correspond to those with notable spikes in movement. Bad volumes were replaced by the average of the volumes on either side.

Statistical modeling & inference

Model type and settings

Linear regression with L2-regularization. To form the design matrix, finite impulse response filters with four bins were placed at delays 2-4, 4-6, 6-8 , and 8-10 s after stimulus onset. The regularization parameter, A, was selected using ten-fold cross-validation within the estimation run data. Specifically, for each value of lambda, each model was fit on 9/10ths of the estimation data. Using the weights estimated, voxel-wise BOLD time-series were predicted for the remaining 1/10th of the data. This was repeated until all of the estimation data had been included once in the held out segment. Concatenating the 10 predicted data segments resulted in a predicted time-series for the entire estimation dataset for each voxel. This complete predicted time-series was correlated with the actual recorded BOLD time-series and the single A value which produced the highest mean correlation value across all voxels was selected. To establish goodness-of-fit, prediction accuracy scores for each voxel were estimated using the validation data by calculating the Pearson correlation between the actual and predicted validation run BOLD timeseries on a voxel by voxel basis.

Effect(s) tested

The voxel-wise model weights for each feature of each model estimate the effect of that feature on that voxel's BOLD timeseries. Prediction scores estimate the total proportion of variance explained by each model within each voxel. The models fit are detailed in Methods.

Specify type of analysis: ☐ Whole brain ☐ ROI-based ☒ Both

Anatomical location(s)

Early visual regions V1-V4 were defined using the retinotopic mapping data. In addition, a simple semantic model with 8 categories (Faces, Bodies, Body Parts, Multiple People, Animals, Food, Objects, Scenes) was fit to the estimation data and used to identify the following functional landmarks on each subject's flat map: RSC, Retrosplenial Complex; OPA, Occipital Place area; LO, Lateral Occipital cortex; pSTS, Posterior Superior Temporal Sulcus; EBA, Extrastriate Body Area; OFA, Occipital Face Area; FFA, Fusiform Face Area; PPA, Parahippocampal Place Area; ATFP, Anterior Temporal Face Patch. We also label the following sulci: IPS, Intraparietal Sulcus; STS, Superior Temporal Sulcus; ITS — Inferior Temporal Sulcus; CoS, Collateral Sulcus; Post-CS, Post Central Sulcus; CS, Central Sulcus; SF, Sylvian Fissure. Note, these ROIs are only for orientation of the viewer and were not used to constrain any of our analyses.

Additionally, several control analyses were conducted using different voxel-selection criteria, as follows:

Non-EVC OTC. Retinotopic mapping (using both rotating wedges and contracting and expanding circles) was used to define early visual cortex (EVC, namely V1-V4) for each participant.

Orbital frontal cortex (OFC): An OFC ROI was created using 4 structural ROIs defined in the AAL template (Tzourio-Mazoyer et al., 2002; Rolls, Joliot, & Tzourio-Mazoyer, 2015), namely the superior, middle, inferior and medial OFC in the bilateral hemispheres. These AAL ROIs were back-projected from MNI space to subject anatomical space using a non-linear transformation {spatial normalization from SPM8}, and then into subject functional space using a linear 12-dimensional affine transformation (spatial co-registration from SPM8).

Non-OFC frontal cortex: This ROI was created by using the following 5 AAL template structural ROIs: superior, middle, and superior medial frontal regions, as well as frontal inferior operculum and frontal inferior triangularis. These ROIs were back-projected into subject functional space in the same manner as the OFC ROI.

Statistic type for inference

(See Eklund et al. 2016)

Voxel-wise explained variance

Correction

FDR correction was obtained via the Benjamini-Hochberg procedure.

Models & analysis

n/a	Involved in the study
☒	☐ Functional and/or effective connectivity
☒	☐ Graph analysis
☐	☒ Multivariate modeling or predictive analysis

Multivariate modeling and predictive analysis

The independent variables for each model were the image features specified for each model. Features were extracted from each image as specified in Methods. Feature weights were estimated using L2-regularized linear regression. Dimensionality reduction was conducted on feature weights of voxels that were significantly predicted by the CSVA model using principal components analysis. Explained variance was compared to chance using permutation testing. The resulting PCs were interpreted by correlation with hypothetical dimensions. Correlations were evaluated using permutation testing of the correlation coefficient. OTC tuning, as captured by CSVA model PCs was used to predict behavioral responses to the images viewed. This prediction performance was compared with that of alternative models. Per image PC scores were entered into regression analyses to predict behavioral responses for each image (see Methods) and leave-one-out cross-validation (LOOCV) was used to calculate the amount of variance in behavioral responses explained.
